# *Arabidopsis* Iron Superoxide Dismutase FSD1 Protects Against Methyl Viologen-Induced Oxidative Stress in a Copper-Dependent Manner

**DOI:** 10.3389/fpls.2022.823561

**Published:** 2022-03-11

**Authors:** Pavol Melicher, Petr Dvořák, Yuliya Krasylenko, Alexey Shapiguzov, Jaakko Kangasjärvi, Jozef Šamaj, Tomáš Takáč

**Affiliations:** ^1^Department of Biotechnology, Faculty of Science, Palacký University Olomouc, Olomouc, Czechia; ^2^Organismal and Evolutionary Biology Research Programme, Faculty of Biological and Environmental Sciences, Viikki Plant Science Centre, University of Helsinki, Helsinki, Finland; ^3^Production Systems Unit, Natural Resources Institute Finland (Luke), Piikkiö, Finland; ^4^Institute of Plant Physiology, Russian Academy of Sciences, Moscow, Russia

**Keywords:** FSD1, *Arabidopsis*, methyl viologen, proteomics, copper, ferredoxin, oxidative stress, superoxide dismutase

## Abstract

Iron superoxide dismutase 1 (FSD1) was recently characterized as a plastidial, cytoplasmic, and nuclear enzyme with osmoprotective and antioxidant functions. However, the current knowledge on its role in oxidative stress tolerance is ambiguous. Here, we characterized the role of FSD1 in response to methyl viologen (MV)-induced oxidative stress in *Arabidopsis thaliana*. In accordance with the known regulation of *FSD1* expression, abundance, and activity, the findings demonstrated that the antioxidant function of FSD1 depends on the availability of Cu^2+^ in growth media. *Arabidopsis fsd1* mutants showed lower capacity to decompose superoxide at low Cu^2+^ concentrations in the medium. Prolonged exposure to MV led to reduced ascorbate levels and higher protein carbonylation in *fsd1* mutants and transgenic plants lacking a plastid FSD1 pool as compared to the wild type. MV induced a rapid increase in FSD1 activity, followed by a decrease after 4 h long exposure. Genetic disruption of *FSD1* negatively affected the hydrogen peroxide-decomposing ascorbate peroxidase in *fsd1* mutants. Chloroplastic localization of FSD1 is crucial to maintain redox homeostasis. Proteomic analysis showed that the sensitivity of *fsd1* mutants to MV coincided with decreased abundances of ferredoxin and photosystem II light-harvesting complex proteins. These mutants have higher levels of chloroplastic proteases indicating an altered protein turnover in chloroplasts. Moreover, *FSD1* disruption affects the abundance of proteins involved in the defense response. Collectively, the study provides evidence for the conditional antioxidative function of FSD1 and its possible role in signaling.

## Introduction

Photosynthetic light reactions are accompanied by the formation of reactive oxygen species (ROS) (Pospíšil, 2016; Foyer, 2018). Superoxide anion radicals (O_2_^⋅−^) and singlet oxygen (^1^O_2_) are the primary ROS, while O_2_^⋅−^ may be converted to other ROS, such as hydrogen peroxide (H_2_O_2_) or hydroxyl radical (⋅OH) (Waszczak et al., 2018). Under normal conditions, most chloroplastic O_2_^⋅−^ is produced on the acceptor site of photosystem I (PSI) (Takahashi and Asada, 1988; Ivanov et al., 2018). Ferredoxin (Fd), the last electron acceptor of PSI, has been reported to produce O_2_^⋅−^; however, the reduction of O_2_ by Fd is highly dependent on available NADP^+^ and is lower than the contribution of O_2_ reduction by membrane-bound photosynthetic electron transport chain components (Kozuleva and Ivanov, 2016).

Methyl viologen (1,1′-dimethyl-4,4′-bipyridinium dichloride; MV), also known as paraquat, is a rapidly acting, non-selective, ROS-inducing herbicide. Its toxicity is exerted by catalyzing light-dependent electron transfer from PSI to O_2_, resulting in O_2_^⋅−^ production (Chia et al., 1982; Hawkes, 2014). With a midpoint redox potential (Em) of −446 mV, the divalent MV cation (MV^2+^) readily accepts a single electron, resulting in the formation of a reduced cation radical (MV^+⋅^). The reaction most probably occurs in PSI; however, the specific site from which MV^2+^ accepts the electron is unclear. It is suggested that either Fd, PSI, or both might be the donors of electrons for MV^2+^ (Hawkes, 2014). Shortly after MV^+⋅^ is produced, it is oxidized by O_2_ to form O_2_^⋅−^. In addition, MV^2+^ is regenerated and can be reduced to MV^+⋅^ again, thus repeating the entire O_2_^⋅−^ evolution cycle (Farrington et al., 1973; Babbs et al., 1989). Within hours of application, the ROS produced by MV can overcome the cellular protective mechanisms and cause severe damage (Hawkes, 2014). Owing to these characteristics, MV is widely used in plant science as an oxidative stress-inducing agent.

MV affects various plant processes. It can cause rapid membrane damage due to lipid peroxidation and induce cell death (Suntres, 2002; Chen and Dickman, 2004). MV-triggered ROS significantly affect photosynthesis, leading to oxidation of the photosystem II (PSII) core protein D1 (Krieger-Liszkay et al., 2011) or inhibition of D1 translation (Nishiyama et al., 2006). Moreover, H_2_O_2_ can damage the water-splitting complex of PSII (Song et al., 2006) and reduce the maximal photochemical efficiency of PSII (Fv/Fm) (Wong, 2000; Li et al., 2010; Krieger-Liszkay et al., 2011; Iriel et al., 2014). In addition, photosynthetic ROS have been demonstrated to have signaling functions, being inevitable for the expression of genes involved in wounding and pathogen attack (Sewelam et al., 2014).

Plants have evolved an efficient antioxidant defense against O_2_^⋅−^ (Dvořák et al., 2021b). Superoxide dismutase (SOD; EC 1.15.1.1) is an antioxidant metalloenzyme that represents the first line of defense against ROS (McCord et al., 1971; Fridovich, 1978; Foyer and Noctor, 2005). SODs catalyze the dismutation of O_2_^⋅−^ to O_2_ and less reactive H_2_O_2_. Several SODs, such as FeSOD, MnSOD, and Cu/ZnSOD, are found in plants having metal cofactors in their active sites. Three genes encoding FeSOD (*FSD1*, *FSD2*, and *FSD3*), one encoding MnSOD (*MSD1*), and three encoding Cu/ZnSOD (*CSD1*, *CSD2*, and *CSD3*) (Kliebenstein et al., 1998; Pilon et al., 2011) have been identified in the *Arabidopsis* genome. They are compartmentalized in mitochondria (MSD1), peroxisomes (CSD3), nuclei (FSD1), cytoplasm (CSD1, FSD1), chloroplast stroma (FSD1), or associated with chloroplast thylakoids (CSD2, FSD2, and FSD3) (Kliebenstein et al., 1998; Morgan et al., 2008; Myouga et al., 2008; Dvořák et al., 2021a). Thus, all *Arabidopsis* FSD isoforms are localized in the chloroplast, whereas FSD1 shows also localization in the cytosol and nucleus. FSD2 and FSD3 proteins are tightly attached to the stromal side of thylakoid membranes and form heterocomplexes in the chloroplast nucleoids. They possibly prevent DNA damage caused by O_2_^⋅−^, as indicated by the physical interaction of FSD3 with the plastid envelope DNA-binding protein (Myouga et al., 2008). Both *fsd2* and *fsd3* mutants show a higher accumulation rate of O_2_^⋅−^ after incubation in the dark, while *fsd1* shows O_2_^⋅−^ levels similar to the wild type (WT; Myouga et al., 2008). Unlike *fsd1*, both *fsd2* and *fsd3* exhibit a high sensitivity to enhanced illumination (Gallie and Chen, 2019). In contrast, another study showed that *fsd1* mutants exhibit hypersensitivity to MV-induced oxidative stress (Dvořák et al., 2021a). Heterologous overexpression of *Arabidopsis FSD1* in tobacco and maize resulted in the increased tolerance to MV. In maize, it also increased growth rates (Van Camp et al., 1996; Van Breusegem et al., 1999). Altogether, these genetic studies have questioned the role of *FSD1* in oxidative stress tolerance.

The main factors affecting *FSD1* expression, abundance, and enzyme activity are the availability of Fe^2+^ (Waters et al., 2012), Cu^2+^ (Cohu et al., 2009; Yamasaki et al., 2009), nitrogen (Mermod et al., 2019), and sucrose (Dugas and Bartel, 2008). *FSD1* expression and abundance increase with low Cu^2+^ availability, in parallel with decline of *CSD1* and *CSD2* expression, when Cu^2+^ is redirected into housekeeping proteins and compounds such as plastocyanin and cytochrome c oxidase (Burkhead et al., 2009; Cohu et al., 2009). In contrast, under Cu^2+^ sufficiency, *CSD1* and *CSD2* expression is enhanced, while *FSD1* expression drops to minimal levels (Cohu et al., 2009; Yamasaki et al., 2009). The well-characterized transcription factor squamosa promoter-binding protein-like 7 (SPL7) is considered a major component in the regulation of Cu^2+^ deficiency responses that tightly controls the expression of the aforementioned SODs in a Cu^2+^-dependent manner (Cohu et al., 2009; Yamasaki et al., 2009). However, the current knowledge on FSD1 role under different Cu^2+^ availability conditions in oxidative stress tolerance is ambiguous. Here, our objective was to address this role and gain insight into the mechanisms of FSD1-dependent responses of *Arabidopsis* to MV. The contribution of FSD1 to the regulation of signaling by photosynthetic ROS is discussed as well.

## Materials and Methods

### Plant Material and Growth Conditions

Mature seeds of *Arabidopsis thaliana* ecotype Col-0 (WT), *fsd1-1*, and *fsd1-2* mutants as well as *fsd1-1* mutants expressing *proFSD1::FSD1:GFP* (FSD1-GFP) or *proFSD1::GFP:FSD1* (GFP-FSD1) (Dvořák et al., 2021a) were surface-sterilized and placed on a half-strength Murashige and Skoog (MS; Murashige and Skoog, 1962) medium, prepared using MS basal salt mixture (cat. n. M0221, Duchefa, Haarlem, Netherlands). For Walz imaging pulse-amplitude-modulation (PAM) measurements, phenotypic analyses, and the examination of Cu^2+^-dependent abundances, and activities of FSD1 and CSDs, a solid half-strength MS medium was prepared manually with increasing concentrations of Cu^2+^ by applying 0–3 μM CuSO_4_⋅5H_2_O. Seeds on the plates were stratified at 4°C for 2 days to synchronize germination. Seedlings were grown at 21°C and 70% humidity under a 16 h light/8 h darkness photoperiod with a photosynthetic photon flux (PPF) of 120 μmol⋅ m^−2^⋅s^–1^ in an environmental chamber (Weiss Technik, Grand Rapids, MI, United States) provided by cool white fluorescent linear tube light sources (Philips Master TL-D Reflex 36 W, light flow 3350 lm, light efficiency 93 lm⋅W^–1^) for a maximum of 14 days. For dark treatment, Petri dishes with 14 days-old plants were fully covered with aluminum foil and cultivated for 2 days in environmental chamber. Control plants were grown under normal light regime (16 h light/8 h darkness) during this period.

For phenotypic analysis, seeds of mutant, transgenic and WT lines were germinated on a half-strength MS medium with different concentrations of Cu^2+^ (0.01, 0.1, and 2 μM) and grown for 5 days. Five-day-old seedlings were transferred to half-strength MS medium with the same concentrations of Cu^2+^ as before, supplemented with 2 μM MV. The percentage ratio of green plants was counted at the 5^th^ day after the transfer. These experiments were performed in triplicate (120 examined seedlings in total). Statistical evaluation of obtained data was carried out by one-way ANOVA test with *post hoc* Tukey HSD test available at an online web statistical calculator^1^. This method was also used for statistical evaluation of results obtained in all subsequent experiments if not mentioned otherwise. The graphical plots were prepared using Microsoft Excel and finalized using Microsoft PowerPoint software.

### Pulse-Amplitude-Modulation Chlorophyll Fluorescence Imaging

The plates with 10-day-old seedlings were loaded with 3 μM MV dissolved in liquid half-strength MS supplemented with 0.05% Tween 20, kept overnight in darkness, and on the next day used for fluorometric measurements of PSII inhibition using the IMAGING-PAM fluorometer (Walz, Effeltrich, Germany) according to a previously published protocol (Shapiguzov et al., 2019). In brief, the seedlings were subjected to 1 h pulses of actinic light, each followed by a 20 min long dark adaptation period and then a saturating light pulse to measure Fv/Fm. The analysis was performed in triplicate.

### MV Treatment for Biochemical and Proteomic Analyses

At least six seedlings of each line, grown vertically for 14 days, were transferred from solid media to 6-well microtiter plates. Each well contained 7 ml of liquid half-strength MS medium supplemented with 1 μM MV. For the mock control, distilled water was used instead of MV, and seedlings were incubated under the same conditions as before. The MV effect was potentiated by an enhanced illumination regime (180 μmol⋅m^–2^⋅s^–1^). The incubation time ranged from 30 min to 8 h. To ensure that circadian oscillations of *FSD1* expression had no impact on the results of analyses, all treatments were terminated at the same time in the day (6 p.m.). After incubation, the seedlings were gently wiped with filter papers to remove excess media and immediately frozen in liquid nitrogen and stored at −80°C.

### Protein Extraction for Biochemical Analyses

The frozen plant material was crushed in liquid nitrogen to a fine powder using a mortar and pestle and transferred to Eppendorf tubes. The powder (100 mg) was resuspended and homogenized with 200 μl extraction buffer containing 50 mM sodium phosphate (pH 7.8), 10% (v/v) glycerol, and 2 mM ascorbate. The tubes were placed on ice for 30 min and occasionally vortexed. Subsequently, the extract was centrifuged at 13 000 × *g* for 20 min at 4°C, and the protein concentration of the supernatant was measured according to the Bradford method (Bradford, 1976). The native protein extract was used for the in-gel detection of SOD and spectrophotometric measurement of ascorbate peroxidase (APX) activity. For immunoblot analysis of FSD1, CSDs, Fd and ferritin, native extracts were supplemented with four times concentrated Laemmli SDS buffer (LB) at a 3:1 ratio (sample: LB) and 5% (v/v) β-mercaptoethanol. For examination of thylakoid APX (tAPX) abundance, proteins were extracted by mixing of the homogenates with RIPA buffer (50 mM Tris–HCl, pH 7.4, 150 mM KCl, 5 mM EGTA, 0.5% (w/v) sodium deoxycholate, 0.1% (v/v) Triton X-100, 0.1% (w/v) SDS, Complete™ EDTA-free Protease Inhibitor Coctail, Roche Diagnostics, Mannheim, Germany) and processed same as samples extracted in sodium phosphate buffer mentioned above. Samples were boiled for 5 min at 95°C.

### Immunoblotting and Analysis of SOD Isozymes

Denatured protein extracts were separated by SDS-PAGE on 10% or 15% (for tAPX detection) TGX Stain-Free™ (Bio-Rad, Hercules, CA, United States) gels prepared according to the manufacturer’s instructions using a Stain-Free FastCast Acrylamide Kit (Bio-Rad). For each sample, 20 μg or 40 μg (tAPX detection) of total protein was loaded onto a gel and separated at 180 V. Proteins were then transferred to a polyvinylidene difluoride (PVDF) membrane (GE Healthcare, Little Chalfont, United Kingdom) in a wet tank unit (Bio-Rad) at a constant current of 230 mA for 1.5 h on ice. To ensure a successful protein transfer, the membrane was documented by ChemiDoc MP Imaging System (Bio-Rad) using the “Stain-Free Blot” protocol in Image Lab software (Bio-Rad). The membrane was blocked with 5% (w/v) Blotting-Grade Blocker (Bio-Rad) in Tris-buffered saline with Tween-20 (TBS-T; 100 mM Tris–HCl, 150 mM NaCl, pH 7.6, 0.1% (v/v) Tween-20) overnight and subsequently incubated with anti-FeSOD (AS06125, diluted 1:3,000), anti-CSD2 (AS06170, diluted 1:2,000), anti-APX (AS08368, diluted 1:5,000), anti-Ferritin (AS10674, diluted 1:3,000), and anti-Ferredoxin 2 (AS204433, diluted 1:3,000) primary antibodies (Agrisera, Vännäs, Sweden) in TBS-T and 1% (w/v) Blotting-Grade Blocker at 4°C overnight. The membrane was washed five times with TBS-T for 10 min. Afterward, it was incubated with horseradish peroxidase-conjugated goat anti-rabbit IgG secondary antibody (Santa Cruz Biotechnology, Santa Cruz, CA, United States), diluted 1:5,000 in TBS-T containing 1% (w/v) BSA for 1.5 h at room temperature. After five washing steps in TBS-T, the membrane was incubated with Clarity Western ECL substrate (Bio-Rad) for 2 min, and chemiluminescence was detected using ChemiDoc MP Imaging System using the “Chemi” protocol in Image Lab software.

For SOD isozyme detection, native protein extracts were separated on 10% native PAGE gel at a constant 20 mA/gel. The visualization of isozymes was performed as described previously (Takáč et al., 2014). The gel was imaged using Image Scanner III (GE Healthcare) and ChemiDoc MP Imaging System.

For both activity and immunoblot analyses, band optical densities were measured using ImageJ software (Schneider et al., 2012). All analyses were performed in triplicate.

### Spectrophotometric Measurement of Ascorbate and APX Activity

Ascorbate concentration was measured in WT, *fsd1* mutants and GFP-FSD1 line subjected to MV treatment for 30 min, 1 and 4 h as previously described (Gillespie and Ainsworth, 2007). APX activity was examined in native protein extracts containing 10 μg protein, by measuring the H_2_O_2_-dependent oxidation of ascorbic acid at 290 nm (Amako et al., 1994). Absorbance was detected for 5 min at 10 s intervals. A molar extinction coefficient of 2.8 mM^–1^⋅cm^–1^ was used to calculate the ascorbic acid content during the experiments. Measurements were performed in three biological replicates.

### Histochemical Detection of O_2_^⋅−^

The O_2_^⋅−^ was visualized in dark-incubated seedlings by histochemical staining using nitroblue tetrazolium (NBT) (Takáč et al., 2014). NBT-stained seedlings were transferred to a storage mixture containing 20% (v/v) glycerol and 80% (v/v) ethanol before loading on glass slides. Whole leaves were then transferred to a glass slide in a drop of 100% glycerol and covered with a coverslip. Imaging of the stained leaves was performed using Image Scanner III. Formazan precipitate intensity was quantified using ImageJ as a mean intensity of specific signal in whole leaves. In total, 30 true leaves were used for staining intensity evaluation.

### Detection of Carbonylated Proteins

Plant material was homogenized in liquid nitrogen and proteins were extracted in 50 mM HEPES (pH 7.5) containing 75 mM NaCl, 1 mM EGTA, 1 mM NaF, 10% (v/v) glycerol, 50 mM DTT, Complete™ EDTA-free Protease Inhibitor Cocktail and PhosSTOP™ (Roche). To analyze carbonylated amino acid residues, a commercial OxyBlot kit (cat n. S7150, Merck KGaA, Darmstadt, Germany) was used. Sample preparation, processing, and immunoblotting were performed according to the manufacturer’s instructions. Signals were visualized by chemiluminescence using the ChemiDoc MP imaging system. The analysis was performed in two biological replicates.

### Proteomic Analysis

Plants (WT, *fsd1-1*, and *fsd1-2* mutants) treated with 1 μM MV for 8 h were homogenized in liquid nitrogen, and the proteins were extracted by phenol extraction (Takáč et al., 2017). Mock-and MV-treated samples were analyzed in triplicate. Proteins were digested by in-solution digestion using sequencing-grade modified trypsin (Takáč et al., 2017). Digested peptides were resuspended in 15 μL formic acid, and 5 μL of the suspension was used for the analysis.

The LC-ESI-MS/MS analyses were performed on a nanoflow HPLC system (Easy-nLC1200, Thermo Fisher Scientific, Bremen, Germany) coupled to an Orbitrap Fusion Lumos mass spectrometer (Thermo Fisher Scientific) equipped with a nano-electrospray ionization source. Peptides were first loaded onto a trapping column and subsequently separated on a 15 cm C18 column (75 μm × 15 cm, ReproSil-Pur 5 μm 200 Å C18-AQ, Dr. Maisch HPLC GmbH, Ammerbuch-Entringen, Germany). The mobile phase consisted of water with 0.1% formic acid (solvent A) and acetonitrile/water [80:20 (v/v)] with 0.1% formic acid (solvent B). Peptides were eluted with a linear 110 min gradient from 5 to 21% solvent B in 62 min and then to 36% solvent B in 110 min, followed by a wash stage with 100% solvent B. MS data were acquired automatically using Thermo Xcalibur 4.1. software (Thermo Fisher Scientific). An information-dependent acquisition method consisted of an Orbitrap MS survey scan with a mass range of 300–1750 m/z followed by HCD fragmentation for the most intense peptide ions.

Data files were searched for protein identification using Proteome Discoverer 2.3 software (Thermo Fisher Scientific) connected to an in-house server running the Mascot 2.7.0 software (Matrix Science, Boston, MA, United States). Data were searched against the SwissProt database (version 2019_11) using the *A. thaliana* taxonomy filter. The following parameters were used: static modifications: carbamidomethyl (C)*, variable modifications: oxidation (M), acetyl (protein N-term), peptide mass tolerance: ± 10 ppm, fragment mass tolerance: ± 0.02 Da, maximum missed cleavages: 2. Methionine oxidation is a common modification during sample processing and is generally included in the search parameters. For quantitation, a minimum of two files containing the same features was obtained, and the unique peptides were used. The precursor abundance was based on the intensity. All peptides were used for normalization. One-way ANOVA (adjusted *p* ≤ 0.05) was used to filter statistically significant results, applied to proteins exhibiting a fold change ≥ of 1.5. Proteins identified by one peptide were excluded from the analysis. Proteins present in all three replicates corresponding to the control proteome and absent in all three replicates of the test proteome were considered unique for the control proteome and vice versa.

#### Bioinformatic Evaluation of Differential Proteomes

Proteins showing different abundances between samples were classified using Gene Ontology (GO) annotation analysis using OmicsBox software (BioBam Bioinformatics, Valencia, Spain). BLAST was performed against the *A. thaliana* NCBI database, permitting 1 BLAST hit. The following parameters were used for annotation: *E* value hit filter 1.0E^–6^; annotation cutoff: 55; GO weight: 5, GO Slim. The iron-sulfur cluster binding proteins from amino acid sequences of the differential proteomes of WT, *fsd1-1*, and *fsd1-2* mutants were predicted using MetalPredator (Valasatava et al., 2016).

## Results

### FSD1 Protects PSII From MV-Induced Inhibition in a Cu^2+^-Dependent Manner

To evaluate the impact of FSD1 on photosynthetic performance upon MV treatment, we monitored the time course of PSII inhibition using chlorophyll fluorescence in intact WT plants, *fsd1* mutants and *fsd1-1* mutant carrying either FSD1-GFP or GFP-FSD1 fusion proteins. A previous study has shown that the N-terminal fusion of GFP to FSD1 disturbs the plastid-targeting motif, and the GFP-FSD1 line lacks the plastidic pool of FSD1. Line harboring FSD1-GFP contains FSD1 in abundance similar to WT. Moreover, the study demonstrated that the abundance and activity of FSD1 in the WT and both complemented lines vary inversely with Cu^2+^ concentration in the growth media. In contrast, the activities of CSD isozymes positively correlate with Cu^2+^ concentration (Dvořák et al., 2021a). The application of a better scaled Cu^2+^ concentration gradient (0, 0.01, 0.1, 0.5, and 2 μM) allowed to define that CSD activity and the abundance of both CSD1 and CSD2 isozymes appear at a concentration of 0.1 μM Cu^2+^ and higher. At this concentration, plants also showed low levels of FSD1 protein abundance as well as enzyme activity, which grow with decreasing Cu^2+^ concentrations (Supplementary Figure 1).

**FIGURE 1:**
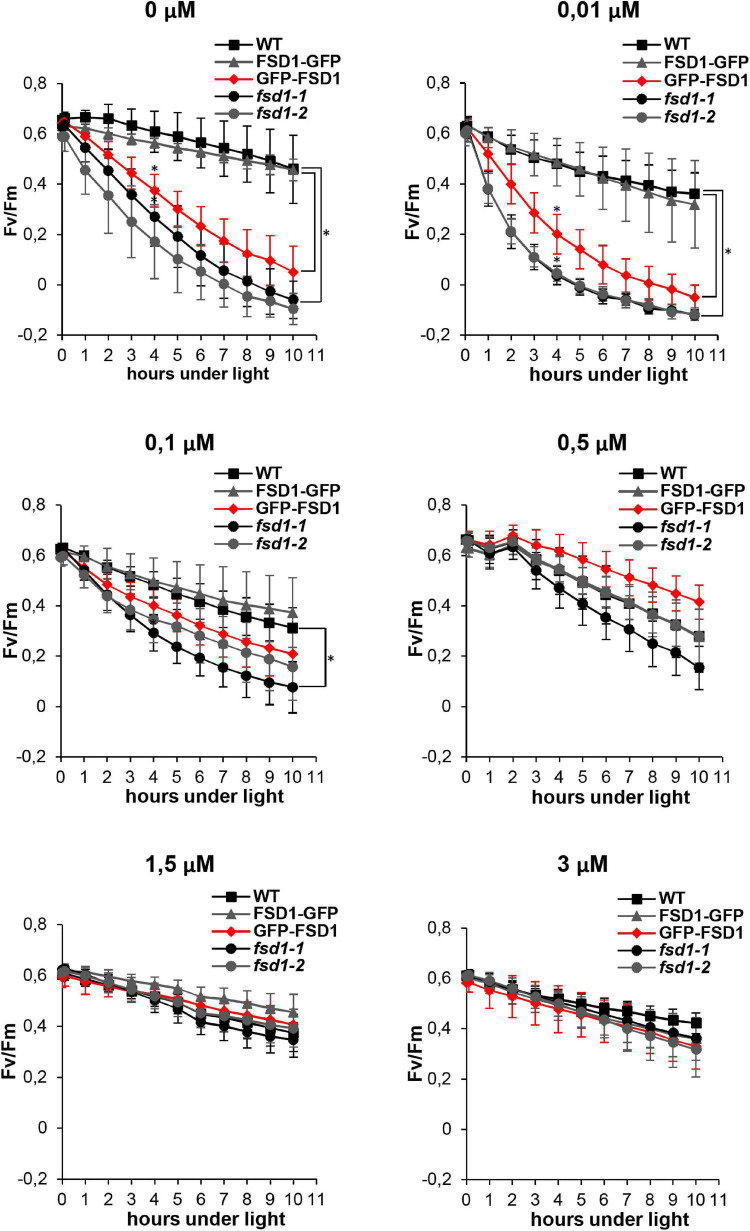
Effect of ROS production induced by methyl viologen (MV) on the PSII activity (Fv/Fm) in wild type (WT), *fsd1* mutants and complemented *fsd1-1* mutants harboring FSD1-GFP or GFP-FSD1 upon different concentrations of Cu^2+^ in the growing media as indicated above the graphs. (mean ± SD, *N* = 9). Asterisks indicate a statistically significant difference between WT and *fsd1-1* or GFP-FSD1 lines as revealed by one-way ANOVA with *post hoc* Tukey HSD test (*p* < 0.05).

Therefore, we estimated the kinetics of PSII inhibition at different Cu^2+^ levels to unravel the effects of FSD1 on photosynthesis. PAM measurements indicated that the sensitivity of the *fsd1* mutants and the GFP-FSD1 line to MV was dependent on Cu^2+^ levels in the growth medium, with the highest rate of PSII inhibition observed upon Cu^2+^ deficiency. At the same time, in the WT and FSD1-GFP line, PSII inhibition was less pronounced and was largely independent of Cu^2+^ concentrations. After 4 h of exposure to light and at a Cu^2+^ concentration of 0.01 μM or lower, the difference between the sensitive and tolerant genotypes was statistically significant (Figure 1). The quantum efficiency of PSII in the GFP-FSD1 line was slightly higher than that of the mutants, but the difference between them was not significant. The difference in PSII inhibition between sensitive and tolerant genotypes was alleviated at higher Cu^2+^ concentrations and became indistinguishable at 0.5 – 3 μM Cu^2+^ (Figure 1). These results suggest that plastidic FSD1 protects the photosynthetic apparatus in *Arabidopsis* against MV only under low Cu^2+^ conditions.

**FIGURE 2:**
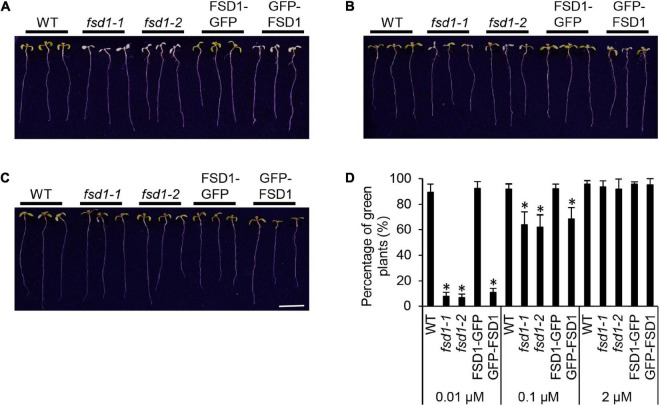
Phenotypic response of wild type (WT), *fsd1* mutants, FSD1-GFP and GFP-FSD1 complemented lines to methyl viologen (MV) at different Cu^2+^ concentrations. **(A–C)** Representative pictures of seedlings documented on 5^th^ day after the transfer to media with 2 μM MV and different Cu^2+^ concentrations (**A**, 0.01 μM; **B** 0.1 μM; **C**, 2 μM). Bar represents 1 cm. **(D)** Relative quantification of fully green seedlings from all examined plants. Asterisks indicate a statistically significant difference at a *p* < 0.05 as determined by one-way ANOVA with *post hoc* Tukey HSD test (mean ± SD, *N* = 120).

Subsequent phenotypic examination pointed to the hypersensitivity of mutant seedlings and GFP-FSD1 lines to MV at 0.01 μM Cu^2+^ as demonstrated by decreased viability, as compared to WT and FSD1-GFP line (Figures 2A,D). However, higher Cu^2+^ concentration in the medium (0.1 μM) increased the rate of viable mutant seedlings reaching roughly 60% compared to the WT (Figures 2B,D). At 2 μM Cu^2+^, all mutant and transgenic lines showed a response very similar to WT (Figures 2C,D).

### FSD1 Deficiency Alters the O_2_^⋅−^ Scavenging Capacity in Mutants

**FIGURE 3:**
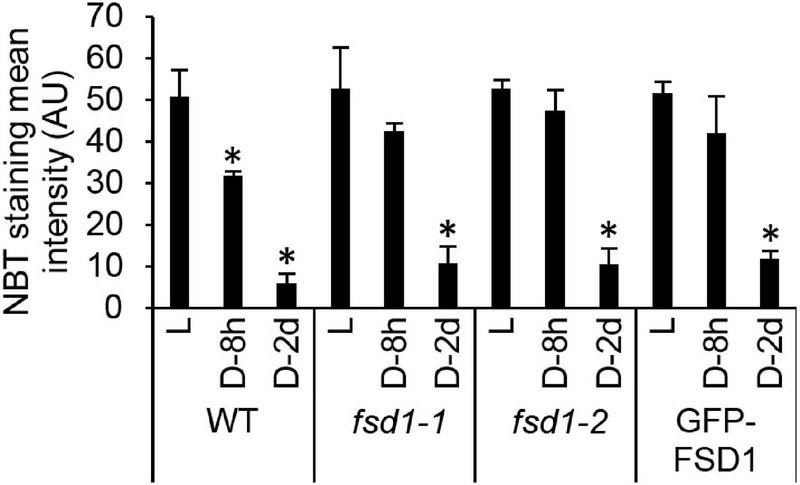
Superoxide production rate in dark incubated seedlings. Semiquantitative analysis of superoxide staining in the true leaves of wild type plants (WT), *fsd1* mutants and GFP-FSD1 line incubated for 2 days (D-2d) and 8 h (D-8h) in darkness or under normal light regime (L) as estimated by quantification of signal intensity obtained by histochemical staining of leaves using nitroblue tetrazolium chloride (NBT). Asterisks indicate a statistically significant difference at a *p* < 0.05 as determined by one-way ANOVA with *post hoc* Tukey HSD test (mean ± SD, *N* = 30 per line). Representative NBT-stained leaf images are shown in Supplementary Figure 2.

In the following experiment, we aimed to analyze the O_2_^⋅−^ scavenging capacity in WT, mutants, and transgenic lines cultivated in darkness for 8 h and 2 days. Histochemical staining of O_2_^⋅−^ using NBT (forming a blue precipitate of formazan after reaction with O_2_^⋅−^) demonstrated a decrease in formazan production in darkness as compared to light-exposed seedlings, the extent of which differed among the studied lines (Figure 3 and Supplementary Figure 2). At both time points, the decrease of staining intensity was more pronounced in WT and FSD1-GFP lines compared to the mutants and GFP-FSD1 lines, suggesting an altered O_2_^⋅−^ scavenging capacity in *fsd1* mutants as well as GFP-FSD1 plants.

### Chloroplast-Localized FSD1 Affects the Ascorbate Content and the Level of Protein Carbonylation Under MV Exposure

**FIGURE 4:**
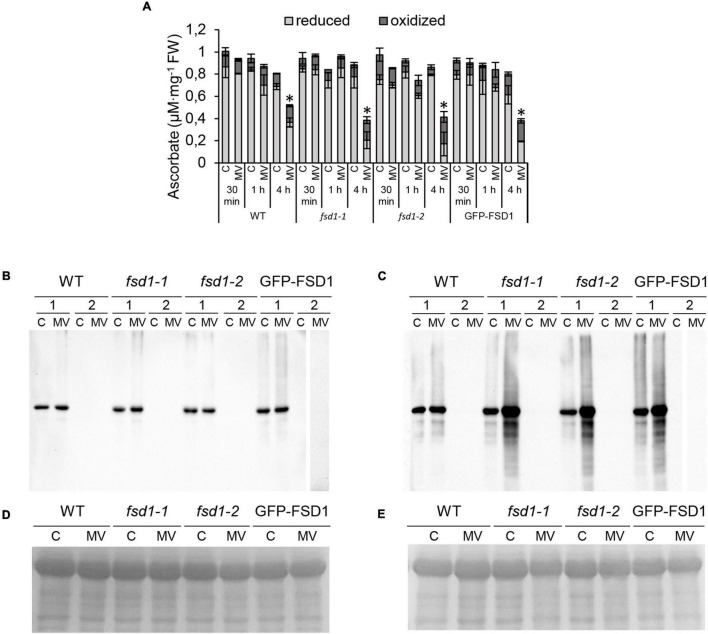
Ascorbate measurement and detection of protein carbonylation in plants exposed to methyl viologen (MV) and controls. **(A)** Quantification of oxidized and reduced ascorbate level in seedlings treated with 1 μM MV for 30 min, 1 h and 4 h. Values are expressed as a concentration of oxidized and reduced ascorbate per mg of fresh weight (FW). Asterisks indicate a statistically significant difference at a *p* < 0.05 as determined by one-way ANOVA with *post hoc* Tukey HSD test (mean ± SD, *N* = 3). **(B,C)** Detection of carbonyl groups in WT, *fsd1-1*, *fsd1-2* and GFP-FSD1 lines after 1 h **(B)** and 4 h **(C)** of MV treatment. Each blot contains protein extracts from mock (lane C) and 1 μM MV treated (lane MV) plants. Each sample was treated either with DNPH oxidation reagent (1) or a control reagent (2) included in the OxyBlot kit. The MV-treated samples of GFP-FSD1 derivatized with control reagent were run on separate gels followed by the same procedure. **(D,E)** Protein gels stained with Coomassie Brilliant Blue G-250 reagent demonstrating the equal amounts of proteins loaded on gel for 1 h **(D)** and 4 h **(E)** treatment. Uncropped, full original images of the blots are documented in Supplementary Figures 3, 4.

To examine better the effect of FSD1 on the oxidative stress response, the levels of ascorbate and its oxidation status were spectrophotometrically estimated in the studied lines. Four-hours-long MV treatment substantially reduced the ascorbate levels in *fsd1* mutants and GFP-FSD1 line, while a less intensive decrease was observed in WT. At this time point MV caused an increase of oxidized ascorbate pool in all lines, while slightly higher ascorbate oxidation was detected in the mutants and GFP-FSD1 line, as compared to WT (Figure 4A).

To monitor the protein oxidation rate, we performed an oxyblot analysis that detected carbonyl groups by immunoblotting using a specific primary antibody (Figures 4B–E and Supplementary Figures 3, 4). One hour of MV treatment did not lead to significant changes in the intensity of the immunoreactive signal (Figure 4B), while genotype-specific changes became apparent after 4 h (Figure 4C). In the WT, we observed only a slight increase in signal intensity. However, a substantial increase in protein oxidation was found in the *fsd1* mutants and the GFP-FSD1 line. These results suggest that the sensitivity of plants to MV concerning the impact on the overall oxidative environment could be higher in the lines in which FSD1 is absent or not localized in plastids.

### The Response of FSD1 and APX to MV Exposure

**FIGURE 5:**
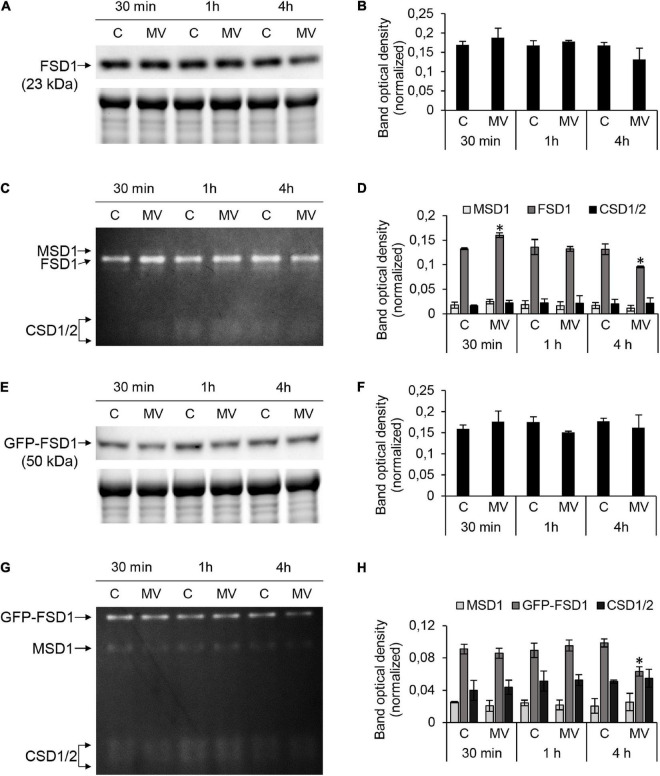
FSD1 abundance and superoxide dismutase (SOD) activity in wild type (WT) and GFP-FSD1 line in response to methyl viologen (MV). **(A,E)** Immunoblots of FSD1 in WT **(A)** and GFP-FSD1 fusion protein in GFP-FSD1 line **(E)**, supplemented with respective controls of protein loading as visualized on Stain-free gels. Each immunoblot contains protein extracts from mock (lane C) and 1 μM MV treated (lane MV) plants. **(B,F)** Quantification of band optical density in **(A,E)**, respectively. **(C,G)** SOD activity staining in WT **(C)** and GFP-FSD1 line **(G)**. Each gel contains protein extracts from mock (lane C) and 1 μM MV-treated (lane MV) plants. **(D,H)** Quantification of band optical density in **(C,G)**, respectively. Blots and gels for each line were prepared on separate membranes. Raw band optical densities in membranes and gels were normalized according to the total density of the specific bands on each individual membrane or gel (mean ± SD, *N* = 3). Asterisks indicate a statistically significant difference between mock control and 1 μM MV treatment in designated time points as calculated by one-way ANOVA with *post hoc* Tukey HSD test (asterisk indicates statistical significance at *p* < 0.05). Uncropped, full original images of the blots and gels are documented in Supplementary Figures 5, 6.

Next, we tested the response of FSD1 to MV treatment. Staining of SOD activity on native polyacrylamide gels allows differentiation among FSD1, MSD1, and Cu/ZnSOD activities (Takáč et al., 2014). Low Cu^2+^ levels in the media led to high FSD1 and low Cu/ZnSOD activities in the examined plants (Figure 5). These findings suggested that the specific response of FSD1 to MV can be determined while minimizing possible redundancy with CSD2 that is localized in plastids as well.

We observed that in WT, FSD1 abundance did not change significantly, although a slight decrease after 4 h of MV treatment was observed (Figures 5A,B and Supplementary Figures 5A,B). FSD1 activity showed significant changes and it increased after 30 min and declined after 4 h (Figures 5C,D and Supplementary Figure 5C). Similar results for FSD1 activity and abundance were observed in the FSD1-GFP line (data not shown). Abundance of GFP-FSD1 did not change substantially throughout the experiment (Figures 5E,F and Supplementary Figures 6A,B). Its activity remained unaffected after 30 min and 1 h and was significantly inhibited after 4 h of MV treatment. It is noteworthy, that decrease in FSD1 activity was more pronounced in GFP-FSD1 line as compared to WT (Figures 5G,H and Supplementary Figure 6C).

Activities of MSD1 and CSD isozymes were not changed throughout the experiment neither in WT, nor in GFP-FSD1 line. The ratio of total activity of CSD isozymes to FSD1 activity in GFP-FSD1 line was higher than in WT plants, due to the lower overall FSD1-GFP activity. We also examined the CSD and MSD1 isozymes activities in *fsd1* mutants. While CSD activities were negligible, activities of MSD1 did not exhibit significant changes during MV treatment (Supplementary Figure 7). These observations indicate that FSD1 activity and abundance are sensitive to MV and that its chloroplast localization is required for this sensitivity. CSDs and MSD1 does not substitute the missing FSD1 activity in *fsd1* mutants.

**FIGURE 6:**
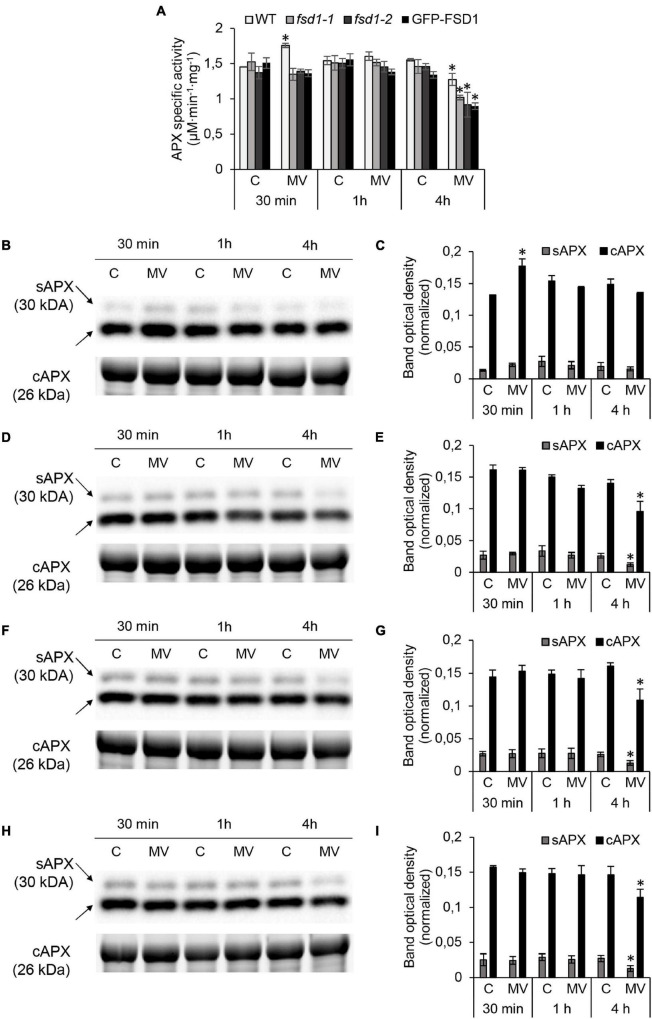
Analysis of ascorbate peroxidase (APX) activity and cytosolic (cAPX) or stromal (sAPX) APX abundances in wild type (WT), *fsd1-1*, *fsd1-2* and GFP-FSD1 lines after methyl viologen (MV) treatment. **(A)** Specific activity of APX measured spectrophotometrically. **(B,D,F,H)** Immunoblots of cAPX in wild type (WT; **B**), *fsd1-1* **(D)**, *fsd1-2* **(F)** and GFP-FSD1 line **(H)**, supplemented with respective controls of protein loading as visualized on Stain-free gels. Each immunoblot contains protein extracts from mock (lane C) and 1 μM MV treated (lane MV) plants. **(C,E,G,I)** Quantification of bands optical density in **(B,D,F,H)**, respectively. Blots for each line were prepared on separate membranes. Raw band optical densities were normalized according to the total density of the respective specific bands on the membrane (mean ± SD, *N* = 3). Asterisks indicate a statistically significant difference between mock and 1 μM MV treated plants in designated time points as calculated by one-way ANOVA with *post hoc* Tukey HSD test (asterisk indicates statistical significance at *p* < 0.05). Uncropped, full original images of the blots and gels are documented in Supplementary Figures 9, 10.

APX is an enzyme with chloroplastic (thylakoid, stroma), cytoplasmic, mitochondrial, and peroxisomal localizations and is responsible for the H_2_O_2_ degradation (Pandey et al., 2017). We analyzed the total APX activity spectrophotometrically and the abundance of stromal (sAPX), tAPX, and cytosolic APX (cAPX) in these lines by immunoblotting. The APX-specific activity in the mock control was similar among the studied lines (Figure 6A). After 30 min of MV treatment, APX activity increased by 21% in WT, while in *fsd1* mutants and GFP-FSD1 line, its activity remained unchanged (*fsd1-2*) or slightly decreased (*fsd1-1* and GFP-FSD1 line) compared to the control. There was no significant difference in the APX activity among the studied lines after 1 h of MV application (Figure 6A). In contrast, all lines showed a substantial decrease in APX activity after 4 h treatment with MV (Figure 6A). In WT, *fsd1-1*, *fsd1-2* mutants, and GFP-FSD1 line, APX activity decreased by 18, 30, 37, and 33%, respectively, as compared to that in the mock control (Figure 6A). These results indicate that the sensitivity of APX activity depends on the presence of FSD1 and its localization to plastids.

Anti-APX primary antibody allows to detect sAPX, tAPX, and cAPX by immunoblotting (Kameoka et al., 2021). For tAPX (Supplementary Figure 8), a different extraction procedure and higher protein load was necessary as for cAPX and sAPX (Figures 6B–I and Supplementary Figures 9, 10). For cAPX, this antibody recognizes two cAPX isoforms (APX1 and APX2) with a 0.5 kDa difference in molecular weight, which comigrate on SDS-PAGE gels. Treatment with MV for 30 min led to a significant increase of cAPX and slight increase of sAPX and tAPX abundances, followed by a slight insignificant decrease of all isozymes after 4 h in WT (Figures 6B,C and Supplementary Figures 8A,B). In contrast, in the *fsd1* mutant lines, abundance of all isoforms did not change as early as 1 h, followed by a substantial decrease after 4 h (Figures 6D–G and Supplementary Figures 8C–F). Similar extent of abundance decline was also observed in the GFP-FSD1 line, showing dependence of APX abundance on the FSD1 plastidial localization (Figures 6H,I and Supplementary Figures 8G,H).

Taken together, MV induced a rapid transient increase in FSD1 and APX activities, as well as FSD1 and APX isoforms abundances. In particular, only plastidial FSD1 was responsive to short-term MV treatment. Prolonged MV exposure led to decreased abundances and activities of both FSD1 and APX, and these effects were more pronounced when FSD1 was missing in chloroplasts.

### Proteomic Analysis of *fsd1* Mutants After MV Treatment

We performed a comparative proteomic analysis of WT, *fsd1-1*, and *fsd1-2* lines exposed to MV for 8 h to better understand the mechanisms underlying higher sensitivity of *fsd1* mutants to MV.

**FIGURE 7:**
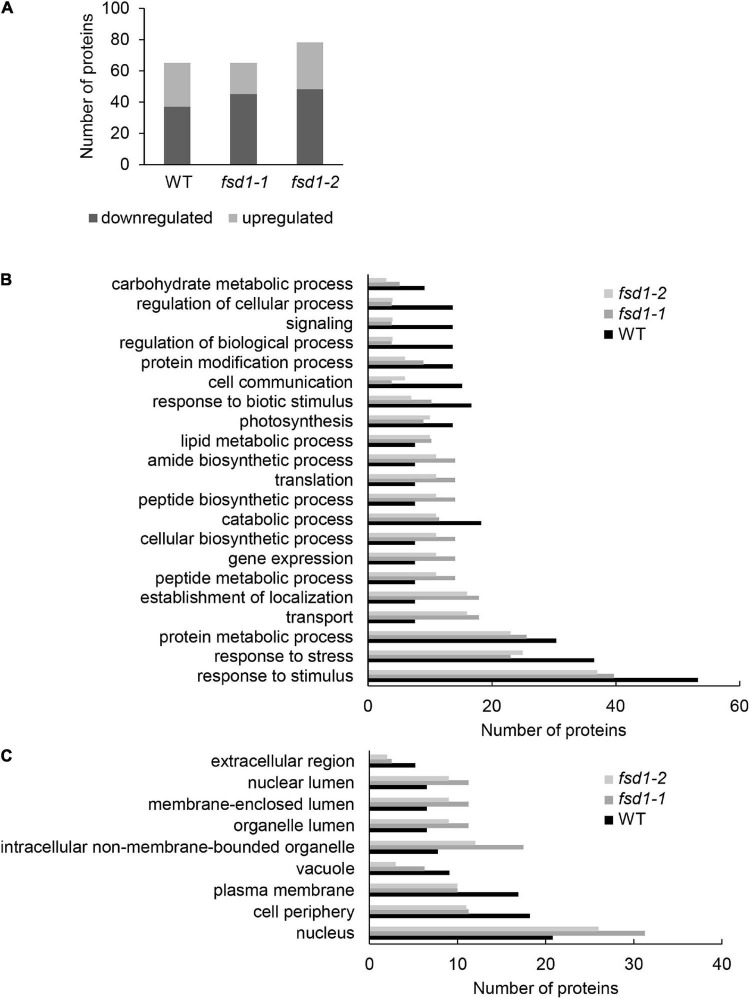
Evaluation of differentially abundant proteins (DAP) found in wild type (WT), *fsd1-1* and *fsd1-2* mutants exposed to 1 μM methyl viologen (MV) for 8 h as compared to mock-treated controls. **(A)** Graph showing the comparison of numbers of upregulated and downregulated DAP. **(B,C)** Graph showing the abundance of gene ontology annotations found in the differential proteomes of the examined lines according to biological process **(B)**, and cell compartment **(C)**. Note, that in panel **(B,C)**, only those annotations, which differ among the lines are included.

Two comparisons were performed. First, for each of the examined lines, we compared the proteomes of MV- and mock-treated plants, and identified 65, 65, and 78 differentially abundant proteins in WT, *fsd1-1*, and *fsd1-2* plants, respectively (Figure 7A and Supplementary Data Sheets 1–3). Second, we compared the proteomes of MV-treated *fsd1* mutants with MV-treated WT plants and found that the differential proteomes contain 59 and 63 proteins in *fsd1-1* and *fsd1-2* mutants (Supplementary Data Sheets 4, 5), respectively. In analogy, the comparison of mock-treated *fsd1* mutants with mock-treated WT resulted in 58 and 61 differentially abundant proteins (Supplementary Data Sheets 6, 7). The information pertinent to protein quantification can be found in the Supplementary Data Sheets 1–7.

We functionally classified the differentially abundant proteins identified by the first comparison using GO annotations (Figures 7B,C and Supplementary Figures 11, 12). MV substantially affected proteins involved in metabolism (i.e., primary, nitrogen compound, and protein metabolism) as well as in the responses to abiotic stimuli and chemicals (Figure 7B). A smaller but significant change was observed in the following GO categories: responses to biotic, endogenous, and light stimuli, cell communication, signaling, gene expression, photosynthesis, and protein modification process (Figure 7B and Supplementary Figure 11). While the differential proteome of WT contained more proteins in categories such as stress response, catabolism, photosynthesis, response to biotic stimuli, cell communication, and signaling, other categories such as transport, peptide metabolic process, gene expression, and translation were more abundant in the mutants (Figure 7B). These findings suggest that FSD1 deficiency in the mutants limits the change in the abundance of proteins involved in stress response, signaling, photosynthesis, and proteins mediating cell-environment communication. Interestingly, MV led to the enrichment of proteins involved in biotic stress responses in the WT, but to a smaller extent in the mutants.

GO annotation according to the cell compartment indicated that MV affected mainly cytoplasmic proteins and proteins localized in the plasma membrane and membranes of chloroplasts (thylakoids), nuclei, mitochondria, and the endomembrane system. Annotations such as nuclei and intracellular non-membrane-bounded organelles were enriched in mutants, while those named plasma membrane, vacuole, and extracellular region were more abundant in WT (Figure 7C and Supplementary Figure 12).

#### *fsd1* Mutants Exhibit More Intensive Deregulation of Photosynthetic Proteins

To gain insight into the possible mechanisms of FSD1-dependent MV tolerance, we focused on proteins involved in light reactions of photosynthesis (Table 1).

**TABLE 1:** Proteins involved in light reactions of photosynthesis.

		Fold change (MV vs. mock control)		
Accession	Name	WT	*fsd1-1*	*fsd1-2*
	**Photosystem II**			
P56778	Photosystem II CP43 reaction center protein		0.625	
P56780	Photosystem II reaction center protein H	1.646		
	**Light harvesting complex II**			
Q9SHR7	Chlorophyll a-b binding protein 2.1, chloroplastic	1.623		
P0CJ48	Chlorophyll a-b binding protein 2, chloroplastic	1.594		
Q9S7W1	Chlorophyll a-b binding protein CP29.3, chloroplastic			0.524
	**Photosystem I**			
P42699	Plastocyanin major isoform, chloroplastic	1.62		
Q9SY97	Photosystem I chlorophyll a/b-binding protein 3-1, chloroplastic	1.607		
Q9SUI7	Photosystem I reaction center subunit VI-1, chloroplastic			1.476
Q9SUI5	Photosystem I reaction center subunit psaK, chloroplastic			1.624
O23344	Ferredoxin C 1, chloroplastic	0.557	0.493	0.483
O04090	Ferredoxin-1, chloroplastic		0.363	0.342
Q39161	Ferredoxin–nitrite reductase, chloroplastic		0.542	0.541
P16972	Ferredoxin-2, chloroplastic		0.309	0.344
Q9C7Y4	Ferredoxin C 2, chloroplastic		0.38	
	**PSII protein turnover**			
O80860	ATP-dependent zinc metalloprotease FtsH 2, chloroplastic	0.568		
Q39102	ATP-dependent zinc metalloprotease FtsH 1, chloroplastic	0.477		

*Note that the empty cell means that the protein was not found in the differential proteome of a respective line.*

**FIGURE 8:**
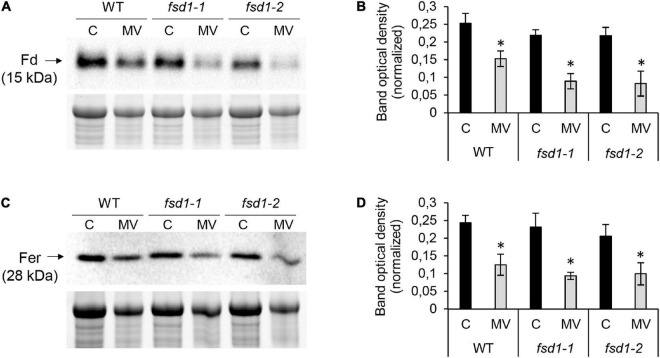
Immunoblotting analysis of ferredoxin (Fd) and ferritins (Fer) in wild type (WT) plants, *fsd1-1* and *fsd1-2* mutants subjected to 8 h long methyl viologen (MV) treatment. **(A,C)** Immunoblots of Fd **(A)** and Fer **(C)** in WT, *fsd1-1* and *fsd1-2*, supplemented with respective controls of protein loading as visualized on Stain-free gels. **(B,D)** Quantification of band optical densities in **A** and **C**, respectively. Each blot contains protein extracts from mock (lane C) and 1 μM MV-treated (lane MV) plants. Raw band optical densities were normalized according to the total density of the specific bands on the membrane (mean ± SD, *N* = 3). Asterisks indicate a statistically significant difference between mock control and 1 μM MV treatment in 8 h as revealed by one-way ANOVA with *post hoc* Tukey HSD test (*p* < 0.05). Uncropped, full original images of blots and gels are documented in Supplementary Figure 13.

The abundance of the chlorophyll *a-b* binding protein isoforms, comprising the light-harvesting complex of PSII (Rantala et al., 2020) increased in WT and decreased in the *fsd1-2* mutant. MV treatment caused an increase in the abundance of PSI components, including the antenna protein LHCA3 and the plastocyanin major isoform in WT plants. A similar increase in PSI component (PSI reaction center subunit VI-1 and subunit psaK) abundance was observed in *fsd1-2* mutants (Table 1). After MV treatment, *fsd1* mutants showed lower levels of PSI reaction center subunits VI-1 and VI-2 as well as plastocyanin major isoform as compared to WT (Supplementary Data Sheets 4, 5). Substantial differences between the WT and mutants were observed in the abundance of Fd isoforms. While MV treatment decreased level of one Fd isoform (C1) in WT, four Fd isoforms were affected in the mutants (Table 1). The levels of Fd1 and Fd2 were lower in the mutants, compared to WT after the MV treatment (Supplementary Data Sheets 4, 5). As revealed by immunoblot analysis, chloroplastic Fd immunoreactive to anti-Fd2 antibody showed reduced abundance in both mutants after MV treatment as compared to WT, thus validating the proteomic results (Figures 8A,B and Supplementary Figures 13A,B).

Fds contain Fe-S clusters as prosthetic groups in their structure. Since Fe-S clusters are known targets of O_2_^⋅−^ (Benov, 2001), we used MetalPredator software to predict the presence of proteins binding Fe-S clusters in the differential proteomes (Table 2). Six, ten, and eight Fe-S cluster-binding proteins were affected in the WT, *fsd1-1*, and *fsd1-2* mutants, respectively. The majority of the predicted proteins showed a decreased abundance in response to MV. As noted above, mutants showed reduced levels of multiple Fd isoforms. Some other proteins, such as 3-isopropylmalate dehydratase large subunit and amidophosphoribosyltransferase 2, were equally underrepresented in all lines. Notably, the CDGSH Fe-S domain-containing protein NEET showed a mutant-specific increase in abundance. These results demonstrate the overall increased sensitivity of Fe-S cluster-binding proteins in *fsd1* mutants.

**TABLE 2:** Proteins predicted to possess Fe-S clusters in the differential proteomes of WT, *fsd1-1* and *fsd1-2* mutants subjected to methyl viologen (MV).

		Fold change (MV vs. mock control)		
Accession	Name	WT	*fsd1-1*	*fsd1-2*
Q9SK50	Protein TIC 55, chloroplastic	1.612		
Q9M591	Magnesium-protoporphyrin IX monomethyl ester [oxidative] cyclase, chloroplastic	1.593		
Q94AR8	3-isopropylmalate dehydratase large subunit, chloroplastic	0.606	0.544	0.534
O23344	Ferredoxin C 1, chloroplastic	0.557	0.493	0.483
P92981	5′-adenylylsulfate reductase 2, chloroplastic	0.482		0.01
Q9STG9	Amidophosphoribosyltransferase 2, chloroplastic	0.392	0.423	0.478
Q39161	Ferredoxin–nitrite reductase, chloroplastic		0.542	0.541
P16972	Ferredoxin-2, chloroplastic		0.309	0.344
Q84JT6	Peptide methionine sulfoxide reductase B9		0.348	
P92980	5′-adenylylsulfate reductase 3, chloroplastic		0.01	
Q9FLI7	CDGSH iron-sulfur domain-containing protein NEET		1.728	1.694
O04090	Ferredoxin-1, chloroplastic		0.363	0.342
Q9C7Y4	Ferredoxin C 2, chloroplastic		0.38	

*Note that the empty cell means that the protein was not found in the differential proteome of a respective line.*

Furthermore, we noticed that MV also affected the iron storage protein ferritin 3 in the WT (Supplementary Data Sheet 1). Proteomic data indicated that ferritin levels were altered more strongly in the mutants because they had lower levels of ferritin 1 in addition to ferritin 3 (Supplementary Data Sheets 4, 5). Immunoblotting analysis using anti-ferritin primary antibody demonstrated that MV treatment caused a more intensive decrease in the abundance of ferritins in the mutants than in WT (Figures 8C,D and Supplementary Figures 13C,D).

#### Deregulation of Ribosomal Proteins, Proteins Involved in Translation and Chloroplastic Protein Quality Control in the Mutants

MV treatment caused deregulations of ribosomal proteins as well as proteins involved in translation and plastid protein quality control (Supplementary Data Sheets 1–3). Four cytosolic ribosomal proteins were downregulated, in addition to two proteins involved in plastid protein translation (putative elongation factor TypA-like SVR3 and threonine-tRNA ligase) in WT (Supplementary Data Sheet 1). Both *fsd1* mutants showed, in addition to downregulated cytosolic ribosomal proteins, also alterations in the abundance of chloroplastic ribosomal proteins, being predominantly upregulated by MV treatment (Supplementary Data Sheets 2, 3). The levels of chloroplastic ribosomal proteins were higher in MV-treated mutants when compared to WT (Supplementary Data Sheets 4, 5). Two ATP-dependent zinc metalloproteases, FtsH1 and FtsH2 were downregulated, by MV in WT (Supplementary Data Sheet 1), but not in *fsd1* mutants. Abundances of FtsH1, 2 and 5 are considerably higher in the MV-treated mutants as compared to WT (Supplementary Data Sheets 4, 5).

## Discussion

### The ROS-Protective Function of FSD1 Is Cu^2+^-Dependent

As mentioned above, the current knowledge about the antioxidant roles of FSD1 is ambiguous. Our analyses indicate that FSD1 plays a protective role in a Cu^2+^-dependent manner. The expression and abundance of FSD1 strictly depend on Cu^2+^ and reach its maximum under Cu^2+^ deficiency (Dvořák et al., 2021a). This dependence is linked to the transcriptional control mechanism of *FSD1* expression. At low Cu^2+^ concentrations, a conserved *cis*-element, present in the promoter sequence of *FSD1*, binds the transcription factor SPL7, which activates *FSD1* expression (Yamasaki et al., 2009). At the same time, SPL7 suppresses the expression of *CSD1* and *CSD2* by upregulating miRNA398, which negatively regulates these two genes (Waters et al., 2012). As shown by this study, the protective role of FSD1 is enhanced with decreasing Cu^2+^ concentration. Therefore, the results explain the recently shown MV hypersensitivity of *fsd1* mutants grown on half-strength MS media (Dvořák et al., 2021a) containing low Cu^2+^ concentration (0.05 μM), and thus the ratio of FSD1 to CSD activity is high. Very likely, the similarities between *fsd1* mutants and WT reported previously (Myouga et al., 2008; Gallie and Chen, 2019) appeared due to the different cultivation system with saturated levels of Cu^2+^. Most likely, the O_2_^⋅−^ generated in chloroplasts is decomposed by the CSD2 isoform under high Cu^2+^ levels. Therefore, we assume that the O_2_^⋅−^ dismutation in chloroplasts is ensured by the strict suborganellar distribution of CSD2 and FSD isoforms and an intricate regulatory mechanism evolved for environments with different Cu^2+^ concentrations.

PAM analysis showed a greater inhibition of PSII in the *fsd1* mutants and the GFP-FSD1 line in response to MV, compared to the WT and FSD1-GFP lines. Interestingly, according to the proteomic data, photosystem II CP43 reaction center protein (CP43), which is a PSII internal antenna protein localized in close proximity to D1 (Kato and Sakamoto, 2009), and proteins that constitute the light-harvesting complex of PSII are sensitive to MV in the *fsd1* mutants but not in the WT, suggesting that FSD1 is necessary to eliminate ROS detrimental to PSII. Of note, similar to D1 protein, CP43 undergoes repair during photodamage, and its degradation rates are higher as compared to other PSII subunits (Nelson et al., 2014). CP43 may also regulate the accessibility of D1 protein to proteolysis by FtsH proteases (Krynická et al., 2015). Indeed, FtsH proteases, which are essential for the turnover of PSII proteins (Kato and Sakamoto, 2009; Malnoë et al., 2014), were differently regulated in WT and the *fsd1* mutants. Considering the specific decrease in PSII proteins in *fsd1* mutants, absence of FSD1 possibly limits the efficient activation of PSII repair cycle. Similar results were obtained in *Synechocystis* cells deficient in H_2_O_2_ scavenging enzymes, such as catalase peroxidase and thioredoxin peroxidase (Nishiyama et al., 2001). FSD1 deficiency also led to a possible activation of a specific protein degradation mechanism under MV treatment. Thus, *fsd1* mutants exhibited an unique upregulation of three isoforms of plant UBX domain-containing protein (PUX3, PUX11 and PUX13). They act as adaptor proteins of cell division control protein CDC48 responsible for the extraction of proteins from their environment for reuse or degradation (Zhang et al., 2021). The precise function of the CDC48-PUX module during oxidative stress remains to be elucidated. Nevertheless, our results suggest that it might be related to the degradation of proteins damaged by MV-induced oxidative stress.

MV-induced ROS are probably responsible for reducing the photochemical efficiency of PSII and alteration of redox homeostasis as demonstrated by ascorbate level measurement and protein carbonylation status. Based on the dynamics of FSD1 activity and disturbed O_2_^⋅−^ scavenging activity, it can be inferred that *fsd1* mutants and GFP-FSD1 plants lack the protective role of plastidic FSD1. Additionally, FSD1 activity did not increase in the line expressing only cytoplasmic and nuclear FSD1, highlighting the role of plastidial FSD1 in the plant oxidative stress response. It has to be noted, that despite the activity of cytoplasmic/nuclear FSD1 is sensitive to oxidative conditions evoked by MV treatment, it seems to be inefficient in antioxidant defense in the absence of the chloroplastic FSD1 pool. The importance of cytoplasmic/nuclear FSD1 becomes more crucial under salt stress conditions (Dvořák et al., 2021a), which are followed by different mechanisms of O_2_^⋅−^ generation (Hossain and Dietz, 2016).

FSD1 is also inevitable for maintaining efficient H_2_O_2_ decomposition by APX in stroma, thylakoids and cytoplasm. The downregulation of APX is most likely a result of highly oxidative conditions after 4 h of MV treatment. In agreement, a previous study showed that MV caused significant oxidative damage to the proteins in *Arabidopsis*, which led to the induction of autophagy and thus to the degradation of these damaged proteins (Xiong et al., 2007). Taken together, we suppose that FSD1 deficiency inhibits the H_2_O_2_-decomposing APX, limits the PSII repair cycle, affects the turnover of PSII proteins, but reinforces cytoplasmic degradation during oxidative stress at limited Cu^2+^ concentrations, most likely by decomposition of O_2_^⋅−^.

### FSD1 Deficiency Increases the Sensitivity of Fds to MV

Oxidative modifications of proteins have been efficiently monitored using redox proteomics approaches (Lennicke et al., 2016; Mock and Dietz, 2016). For example, MV causes the oxidation of a wide range of proteins, including RuBisCO, PSII oxygen-evolving complexes 1 and 23K, PSII oxygen-evolving enhancers PSBQ1 and 2, and glutathione S-transferases in *Arabidopsis* (Muthuramalingam et al., 2013). ROS overaccumulation also leads to destructive effects on proteins affecting their turnover (Song et al., 2006).

Quantitative differential shotgun proteomics efficiently reflects changes in protein abundance (Takáč et al., 2011; Frese et al., 2019). Accordingly, MV in our experimental system led to remarkable changes in *Arabidopsis* WT and *fsd1* proteomes. FSD1 deficiency deregulates Fe-S cluster-binding proteins and proteins involved in Fe-S cluster biogenesis during exposure of plants to MV. Proteins binding to the Fe-S cluster are targeted by O_2_^⋅−^ and H_2_O_2_ with differing velocities. Oxidation of Fe-S clusters occurs more slowly by H_2_O_2_ than by O_2_^⋅−^ (Imlay, 2003). Thus, O_2_^⋅−^ efficiently inhibits the enzymatic activity of Fe-S containing dehydratases, aconitase, transketolase, and other enzymes such as catalase and glutathione peroxidase (Imlay, 2008). The sensitivity of Fe-S cluster binding proteins depends on the stoichiometry of the Fe-S cluster and its exposure to the environment (Imlay, 2006). These findings may explain that some Fe-S cluster-containing proteins showed an increase in abundance in response to MV. The breakdown of the Fe-S cluster enhances the levels of free iron and intensifies the generation of highly reactive ⋅OH by the Fenton reaction (Imlay, 2003). Therefore, the elevated downregulation of Fe-S cluster proteins in the mutants indicates a protective role of FSD1 in Fe-S binding protein stability during oxidative stress.

The most pronounced differences between the *fsd1* mutants and WT were found in the Fd isoforms. Fd is a small 2Fe-2S cluster-containing protein, a stromal electron acceptor in PSI (Hanke and Mulo, 2013). Fds have a substantial impact on redox regulation and antioxidant defense (Hanke and Mulo, 2013). Under oxidative stress, the Fe-S cluster of Fds undergoes disassembly (Camba and Armstrong, 2000) and Fds are downregulated in response to environmental cues (Liu et al., 2013). Its oxidation may control the distribution of electrons in either linear or cyclic electron flow (Hanke and Mulo, 2013). Fd1 and Fd2 are designated as two chloroplastic isoforms in *Arabidopsis*, of which Fd2 constitutes more than 90% of the total leaf Fd complement. Fd1 is believed to be essential for cyclic electron flow (Hanke and Hase, 2008). Our results showed that FSD1 deficiency sensitized multiple Fd isoforms to MV, suggesting that FSD1 is linked to the abundance of Fds and might protect them from oxidative damage.

Ferritins, the iron storage proteins, represent another known O_2_^⋅−^ target. They are important for oxidative stress tolerance, and their downregulation elevates ROS levels (Ravet et al., 2009). O_2_^⋅−^ reductively mobilizes free iron from ferritins, potentiating the oxidative damage caused by ⋅OH generated by the Fenton reaction (Reif, 1992). The increased downregulation of ferritin in the mutants as compared to WT further supports the role of FSD1 in antioxidative defense.

### FSD1 Likely Contributes to Stress Signaling

GO analysis showed that MV treatment altered the abundance of proteins involved in pathogen defense in the WT. Among others, it downregulates the cysteine- and histidine-rich domain-containing protein RAR1, which contributes to plant immunity by supporting the stability of R proteins (Hubert et al., 2009). Moreover, it also reduces the abundance of RPM1-interacting protein 4 (RIN4), which is targeted by multiple bacterial effectors and contributes to the suppression of PAMP-triggered immunity (Toruño et al., 2019). Efficient photosynthesis is vital for plant defense against pathogens (Bechtold et al., 2005). It has been shown that chloroplastic H_2_O_2_ induces the expression of genes involved in responses to wounding and pathogen attack (Sewelam et al., 2014) and may serve as a messenger in plastid-nucleus retrograde signaling (Exposito-Rodriguez et al., 2017). Alterations in biotic stress-responsive proteins by MV treatment were also reported in a previous transcriptomic study (Han et al., 2014). O_2_^⋅−^ generated by MV has a broader role during signaling because it activates a wide range of stress-responsive genes (Scarpeci et al., 2008). Our data, showing downregulation of sinaling proteins in *fsd1* mutants, suggest the role of FSD1 in signaling functions of O_2_^⋅−^, particularly toward the abundance of biotic stress-responsive proteins. Notably, yeast nuclear SOD1 acts as a putative co-transcription factor regulating the signaling roles of O_2_^⋅−^ (Tsang et al., 2014). However, further studies are required to ascertain similar role for *Arabidopsis* FSD1.

## Conclusion

Our results provide several lines of evidence that FSD1 is a crucial component of antioxidant machinery, protecting plants against the detrimental effects of O_2_^⋅−^ under low Cu^2+^ concentrations in the growth substrate. It contributes to sustained levels of Fe-S cluster-binding proteins, particularly Fd isoforms, under MV-induced oxidative stress.

## Data Availability Statement

The original contributions presented in the study are publicly available. This data can be found here: PRIDE repository (Perez-Riverol et al., 2019). Project Name: Proteomic analysis of *Arabidopsis fsd1* mutants in response to methyl viologen. Project accession: PXD028328.

## Author Contributions

PM, PD, YK, and AS conducted the experiments and drafted the manuscript with input from all co-authors. TT, JŠ, and JK revised and edited the manuscript. TT conceived and supervised the project.

## Conflict of Interest

The authors declare that the research was conducted in the absence of any commercial or financial relationships that could be construed as a potential conflict of interest.

## Publisher’s Note

All claims expressed in this article are solely those of the authors and do not necessarily represent those of their affiliated organizations, or those of the publisher, the editors and the reviewers. Any product that may be evaluated in this article, or claim that may be made by its manufacturer, is not guaranteed or endorsed by the publisher.

## Footnotes

1
http://astatsa.com/OneWay_Anova_with_TukeyHSD/

## References

[B1] Amako K. Chen G.-X. Asada K. (1994). Separate assays specific for ascorbate peroxidase and guaiacol peroxidase and for the chloroplastic and cytosolic isozymes of ascorbate peroxidase in plants. *Plant Cell Physiol.* 35 497–504. 10.1093/oxfordjournals.pcp.a078621

[B2] Babbs C. F. Pham J. A. Coolbaugh R. C. (1989). Lethal hydroxyl radical production in paraquat-treated plants. *Plant Physiol.* 90 1267–1270. 10.1104/pp.90.4.1267 16666920 PMC1061880

[B3] Bechtold U. Karpinski S. Mullineaux P. M. (2005). The influence of the light environment and photosynthesis on oxidative signalling responses in plant-biotrophic pathogen interactions. *Plant Cell Environ.* 28 1046–1055. 10.1111/j.1365-3040.2005.01340.x

[B4] Benov L. (2001). How superoxide radical damages the cell. *Protoplasma* 217 33–36. 10.1007/BF01289410 11732335

[B5] Bradford M. M. (1976). A rapid and sensitive method for the quantitation of microgram quantities of protein utilizing the principle of protein-dye binding. *Anal. Biochem.* 72 248–254. 10.1006/abio.1976.9999 942051

[B6] Burkhead J. L. Gogolin Reynolds K. A. Abdel-Ghany S. E. Cohu C. M. Pilon M. (2009). Copper homeostasis. *New Phytol.* 182 799–816. 10.1111/j.1469-8137.2009.02846.x 19402880

[B7] Camba R. Armstrong F. A. (2000). Investigations of the oxidative disassembly of Fe-S clusters in Clostridium pasteurianum 8Fe ferredoxin using pulsed-protein-film voltammetry. *Biochemistry* 39 10587–10598. 10.1021/bi000832+ 10956051

[B8] Chen S. Dickman M. B. (2004). Bcl-2 family members localize to tobacco chloroplasts and inhibit programmed cell death induced by chloroplast-targeted herbicides. *J. Exp. Bot.* 55 2617–2623. 10.1093/jxb/erh275 15475374

[B9] Chia L. S. McRae D. G. Thompson J. E. (1982). Light-dependence of paraquat-initiated membrane deterioration in bean plants. Evidence for the involvement of superoxide. *Physiol. Plant* 56 492–499. 10.1111/j.1399-3054.1982.tb04545.x

[B10] Cohu C. M. Abdel-Ghany S. E. Gogolin Reynolds K. A. Onofrio A. M. Bodecker J. R. Kimbrel J. A. (2009). Copper delivery by the copper chaperone for chloroplast and cytosolic copper/zinc-superoxide dismutases: regulation and unexpected phenotypes in an *Arabidopsis* mutant. *Mol. Plant* 2 1336–1350. 10.1093/mp/ssp084 19969519

[B11] Dugas D. V. Bartel B. (2008). Sucrose induction of *Arabidopsis* miR398 represses two Cu/Zn superoxide dismutases. *Plant Mol. Biol.* 67 403–417. 10.1007/s11103-008-9329-1 18392778

[B12] Dvořák P. Krasylenko Y. Ovečka M. Basheer J. Zapletalová V. Šamaj J. (2021a). *In vivo* light-sheet microscopy resolves localisation patterns of FSD1, a superoxide dismutase with function in root development and osmoprotection. *Plant Cell Environ.* 44 68–87. 10.1111/pce.13894 32974958

[B13] Dvořák P. Krasylenko Y. Zeiner A. Šamaj J. Takáč T. (2021b). Signaling toward reactive oxygen species-scavenging enzymes in plants. *Front. Plant Sci.* 11:618835. 10.3389/fpls.2020.618835 33597960 PMC7882706

[B14] Exposito-Rodriguez M. Laissue P. P. Yvon-Durocher G. Smirnoff N. Mullineaux P. M. (2017). Photosynthesis-dependent H_2_O_2_ transfer from chloroplasts to nuclei provides a high-light signalling mechanism. *Nat. Commun.* 8:49. 10.1038/s41467-017-00074-w 28663550 PMC5491514

[B15] Farrington J. A. Ebert M. Land E. J. Fletcher K. (1973). Bipyridylium quaternary salts and related compounds. V. Pulse radiolysis studies of the reaction of paraquat radical with oxygen. Implications for the mode of action of bipyridyl herbicides. *Biochim. Biophys. Acta* 314 372–381. 10.1016/0005-2728(73)90121-7 4751237

[B16] Foyer C. H. (2018). Reactive oxygen species, oxidative signaling and the regulation of photosynthesis. *Environ. Exp. Bot.* 154 134–142. 10.1016/j.envexpbot.2018.05.003 30283160 PMC6105748

[B17] Foyer C. H. Noctor G. (2005). Oxidant and antioxidant signalling in plants: a re-evaluation of the concept of oxidative stress in a physiological context. *Plant Cell Environ.* 28 1056–1071. 10.1111/j.1365-3040.2005.01327.x

[B18] Frese C. K. van den Toorn H. Heck A. J. R. Mohammed S. (2019). “Quantitative proteomics for differential protein expression profiling,” in *Proteomics for Biological Discovery*, eds Veenstra T. D. Yates J. R. (Hoboken, NJ: John Wiley & Sons, Inc.), 1–27. 10.1002/9781119081661.ch1

[B19] Fridovich I. (1978). Superoxide radicals, superoxide dismutases and the aerobic lifestyle. *Photochem. Photobiol.* 28 733–741. 10.1111/j.1751-1097.1978.tb07009.x 216032

[B20] Gallie D. R. Chen Z. (2019). Chloroplast-localized iron superoxide dismutases FSD2 and FSD3 are functionally distinct in Arabidopsis. *PLoS One* 14:e0220078. 10.1371/journal.pone.0220078 31329637 PMC6645559

[B21] Gillespie K. M. Ainsworth E. A. (2007). Measurement of reduced, oxidized and total ascorbate content in plants. *Nat. Protoc.* 2 871–874. 10.1038/nprot.2007.101 17446888

[B22] Han H.-J. Peng R.-H. Zhu B. Fu X.-Y. Zhao W. Shi B. (2014). Gene expression profiles of *Arabidopsis* under the stress of methyl viologen: a microarray analysis. *Mol. Biol. Rep.* 41 7089–7102. 10.1007/s11033-014-3396-y 25253097

[B23] Hanke G. Mulo P. (2013). Plant type ferredoxins and ferredoxin-dependent metabolism: chloroplast ferredoxins. *Plant Cell Environ.* 36 1071–1084. 10.1111/pce.12046 23190083

[B24] Hanke G. T. Hase T. (2008). Variable photosynthetic roles of two leaf-type ferredoxins in *Arabidopsis*, as revealed by RNA interference. *Photochem. Photobiol.* 84 1302–1309. 10.1111/j.1751-1097.2008.00411.x 18673322

[B25] Hawkes T. R. (2014). Mechanisms of resistance to paraquat in plants. *Pest Manag. Sci.* 70 1316–1323. 10.1002/ps.3699 24307186

[B26] Hossain M. S. Dietz K.-J. (2016). Tuning of redox regulatory mechanisms, reactive oxygen species and redox homeostasis under salinity stress. *Front. Plant Sci.* 7:548. 10.3389/fpls.2016.00548 27242807 PMC4861717

[B27] Hubert D. A. He Y. McNulty B. C. Tornero P. Dangl J. L. (2009). Specific *Arabidopsis* HSP90.2 alleles recapitulate RAR1 cochaperone function in plant NB-LRR disease resistance protein regulation. *Proc. Natl. Acad. Sci. U.S.A.* 106 9556–9563. 10.1073/pnas.0904877106 19487680 PMC2689315

[B28] Imlay J. A. (2003). Pathways of oxidative damage. *Annu. Rev. Microbiol.* 57 395–418. 10.1146/annurev.micro.57.030502.090938 14527285

[B29] Imlay J. A. (2006). Iron-sulphur clusters and the problem with oxygen. *Mol. Microbiol.* 59 1073–1082. 10.1111/j.1365-2958.2006.05028.x 16430685

[B30] Imlay J. A. (2008). Cellular defenses against superoxide and hydrogen peroxide. *Annu. Rev. Biochem.* 77 755–776. 10.1146/annurev.biochem.77.061606.161055 18173371 PMC3057177

[B31] Iriel A. Novo J. M. Cordon G. B. Lagorio M. G. (2014). Atrazine and methyl viologen effects on chlorophyll-a fluorescence revisited-Implications in photosystems emission and ecotoxicity assessment. *Photochem. Photobiol.* 90 107–112. 10.1111/php.12142 23869421

[B32] Ivanov B. N. Borisova-Mubarakshina M. M. Kozuleva M. A. (2018). Formation mechanisms of superoxide radical and hydrogen peroxide in chloroplasts, and factors determining the signalling by hydrogen peroxide. *Funct. Plant Biol.* 45 102–110. 10.1071/FP16322 32291025

[B33] Kameoka T. Okayasu T. Kikuraku K. Ogawa T. Sawa Y. Yamamoto H. (2021). Cooperation of chloroplast ascorbate peroxidases and proton gradient regulation 5 is critical for protecting *Arabidopsis* plants from photo-oxidative stress. *Plant J.* 107 876–892. 10.1111/tpj.15352 34028907

[B34] Kato Y. Sakamoto W. (2009). Protein quality control in chloroplasts: a current model of D1 protein degradation in the photosystem II repair cycle. *J. Biochem.* 146 463–469. 10.1093/jb/mvp073 19451147

[B35] Kliebenstein D. J. Monde R. A. Last R. L. (1998). Superoxide dismutase in *Arabidopsis*: an eclectic enzyme family with disparate regulation and protein localization. *Plant Physiol.* 118 637–650. 10.1104/pp.118.2.637 9765550 PMC34840

[B36] Kozuleva M. A. Ivanov B. N. (2016). The Mechanisms of oxygen reduction in the terminal reducing segment of the chloroplast photosynthetic electron transport chain. *Plant Cell Physiol.* 57 1397–1404. 10.1093/pcp/pcw035 27016099

[B37] Krieger-Liszkay A. Kós P. B. Hideg E. (2011). Superoxide anion radicals generated by methylviologen in photosystem I damage photosystem II. *Physiol. Plant* 142 17–25. 10.1111/j.1399-3054.2010.01416.x 20875060

[B38] Krynická V. Shao S. Nixon P. J. Komenda J. (2015). Accessibility controls selective degradation of photosystem II subunits by FtsH protease. *Nat. Plants* 1 1–6. 10.1038/nplants.2015.168 27251713

[B39] Lennicke C. Rahn J. Heimer N. Lichtenfels R. Wessjohann L. A. Seliger B. (2016). Redox proteomics: methods for the identification and enrichment of redox-modified proteins and their applications. *Proteomics* 16 197–213. 10.1002/pmic.201500268 26508685

[B40] Li F. Wu Q.-Y. Sun Y.-L. Wang L.-Y. Yang X.-H. Meng Q.-W. (2010). Overexpression of chloroplastic monodehydroascorbate reductase enhanced tolerance to temperature and methyl viologen-mediated oxidative stresses. *Physiol. Plant* 139 421–434. 10.1111/j.1399-3054.2010.01369.x 20230481

[B41] Liu J. Wang P. Liu B. Feng D. Zhang J. Su J. (2013). A deficiency in chloroplastic ferredoxin 2 facilitates effective photosynthetic capacity during long-term high light acclimation in *Arabidopsis thaliana*. *Plant J.* 76 861–874. 10.1111/tpj.12341 24118453

[B42] Malnoë A. Wang F. Girard-Bascou J. Wollman F.-A. de Vitry C. (2014). Thylakoid FtsH protease contributes to photosystem II and cytochrome b6f remodeling in *Chlamydomonas reinhardtii* under stress conditions. *Plant Cell* 26 373–390. 10.1105/tpc.113.120113 24449688 PMC3963582

[B43] McCord J. M. Keele B. B. Fridovich I. (1971). An enzyme-based theory of obligate anaerobiosis: the physiological function of superoxide dismutase. *Proc. Natl. Acad. Sci. U.S.A.* 68 1024–1027. 10.1073/pnas.68.5.1024 4995818 PMC389105

[B44] Mermod M. Takusagawa M. Kurata T. Kamiya T. Fujiwara T. Shikanai T. (2019). SQUAMOSA promoter-binding protein-like 7 mediates copper deficiency response in the presence of high nitrogen in Arabidopsis thaliana. *Plant Cell Rep.* 38 835–846. 10.1007/s00299-019-02422-0 31093688

[B45] Mock H.-P. Dietz K.-J. (2016). Redox proteomics for the assessment of redox-related posttranslational regulation in plants. *Biochim. Biophys. Acta (BBA) - Proteins Proteomics* 1864 967–973. 10.1016/j.bbapap.2016.01.005 26784836

[B46] Morgan M. J. Lehmann M. Schwarzländer M. Baxter C. J. Sienkiewicz-Porzucek A. Williams T. C. R. (2008). Decrease in manganese superoxide dismutase leads to reduced root growth and affects tricarboxylic acid cycle flux and mitochondrial redox homeostasis. *Plant Physiol.* 147 101–114. 10.1104/pp.107.113613 18337490 PMC2330298

[B47] Murashige T. Skoog F. (1962). A revised medium for rapid growth and bio assays with tobacco tissue cultures. *Physiol. Plant* 15 473–497. 10.1111/j.1399-3054.1962.tb08052.x

[B48] Muthuramalingam M. Matros A. Scheibe R. Mock H.-P. Dietz K.-J. (2013). The hydrogen peroxide-sensitive proteome of the chloroplast *in vitro* and *in vivo*. *Front. Plant Sci.* 4:54. 10.3389/fpls.2013.00054 23516120 PMC3601327

[B49] Myouga F. Hosoda C. Umezawa T. Iizumi H. Kuromori T. Motohashi R. (2008). A heterocomplex of iron superoxide dismutases defends chloroplast nucleoids against oxidative stress and is essential for chloroplast development in *Arabidopsis*. *Plant Cell* 20 3148–3162. 10.1105/tpc.108.061341 18996978 PMC2613658

[B50] Nelson C. J. Alexova R. Jacoby R. P. Millar A. H. (2014). Proteins with high turnover rate in barley leaves estimated by proteome analysis combined with in planta isotope labeling. *Plant Physiol.* 166 91–108. 10.1104/pp.114.243014 25082890 PMC4149734

[B51] Nishiyama Y. Allakhverdiev S. I. Murata N. (2006). A new paradigm for the action of reactive oxygen species in the photoinhibition of photosystem II. *Biochim. Biophys. Acta* 1757 742–749. 10.1016/j.bbabio.2006.05.013 16784721

[B52] Nishiyama Y. Yamamoto H. Allakhverdiev S. I. Inaba M. Yokota A. Murata N. (2001). Oxidative stress inhibits the repair of photodamage to the photosynthetic machinery. *EMBO J.* 20 5587–5594. 10.1093/emboj/20.20.5587 11598002 PMC125664

[B53] Pandey S. Fartyal D. Agarwal A. Shukla T. James D. Kaul T. (2017). Abiotic stress tolerance in plants: myriad roles of ascorbate peroxidase. *Front. Plant Sci.* 8:581. 10.3389/fpls.2017.00581 28473838 PMC5397514

[B54] Perez-Riverol Y. Csordas A. Bai J. Bernal-Llinares M. Hewapathirana S. Kundu D. J. (2019). The PRIDE database and related tools and resources in 2019: improving support for quantification data. *Nucleic Acids Res.* 47 D442–D450. 10.1093/nar/gky1106 30395289 PMC6323896

[B55] Pilon M. Ravet K. Tapken W. (2011). The biogenesis and physiological function of chloroplast superoxide dismutases. *Biochim. Biophys. Acta (BBA) - Bioenerget.* 1807 989–998. 10.1016/j.bbabio.2010.11.002 21078292

[B56] Pospíšil P. (2016). Production of reactive oxygen species by photosystem II as a response to light and temperature stress. *Front. Plant Sci.* 7:1950. 10.3389/fpls.2016.01950 28082998 PMC5183610

[B57] Rantala M. Rantala S. Aro E.-M. (2020). Composition, phosphorylation and dynamic organization of photosynthetic protein complexes in plant thylakoid membrane. *Photochem. Photobiol. Sci.* 19 604–619. 10.1039/D0PP00025F 32297616

[B58] Ravet K. Touraine B. Boucherez J. Briat J.-F. Gaymard F. Cellier F. (2009). Ferritins control interaction between iron homeostasis and oxidative stress in *Arabidopsis*. *Plant J.* 57 400–412. 10.1111/j.1365-313X.2008.03698.x 18826427

[B59] Reif D. W. (1992). Ferritin as a source of iron for oxidative damage. *Free Radic. Biol. Med.* 12 417–427. 10.1016/0891-5849(92)90091-t 1317328

[B60] Scarpeci T. E. Zanor M. I. Carrillo N. Mueller-Roeber B. Valle E. M. (2008). Generation of superoxide anion in chloroplasts of *Arabidopsis thaliana* during active photosynthesis: a focus on rapidly induced genes. *Plant Mol. Biol.* 66 361–378. 10.1007/s11103-007-9274-4 18158584 PMC2758387

[B61] Schneider C. A. Rasband W. S. Eliceiri K. W. (2012). NIH Image to ImageJ:25 years of image analysis. *Nat. Methods* 9 671–675. 10.1038/nmeth.2089 22930834 PMC5554542

[B62] Sewelam N. Jaspert N. Van Der Kelen K. Tognetti V. B. Schmitz J. Frerigmann H. (2014). Spatial H_2_O_2_ signaling specificity: H_2_O_2_ from chloroplasts and peroxisomes modulates the plant transcriptome differentially. *Mol. Plant* 7 1191–1210. 10.1093/mp/ssu070 24908268

[B63] Shapiguzov A. Vainonen J. P. Hunter K. Tossavainen H. Tiwari A. Järvi S. (2019). Arabidopsis RCD1 coordinates chloroplast and mitochondrial functions through interaction with ANAC transcription factors. *eLife* 8:e43284. 10.7554/eLife.43284 30767893 PMC6414205

[B64] Song Y. G. Liu B. Wang L. F. Li M. H. Liu Y. (2006). Damage to the oxygen-evolving complex by superoxide anion, hydrogen peroxide, and hydroxyl radical in photoinhibition of photosystem II. *Photosynth Res.* 90 67–78. 10.1007/s11120-006-9111-7 17131094

[B65] Suntres Z. E. (2002). Role of antioxidants in paraquat toxicity. *Toxicology* 180 65–77. 10.1016/s0300-483x(02)00382-7 12324200

[B66] Takáč T. Pechan T. Šamaj J. (2011). Differential proteomics of plant development. *J. Proteomics* 74 577–588. 10.1016/j.jprot.2011.02.002 21315196

[B67] Takáč T. Šamajová O. Pechan T. Luptovčiak I. Šamaj J. (2017). Feedback microtubule control and microtubule-actin cross-talk in *Arabidopsis* revealed by integrative proteomic and cell biology analysis of KATANIN 1 mutants. *Mol. Cell Prot.* 16 1591–1609. 10.1074/mcp.M117.068015 28706004 PMC5587860

[B68] Takáč T. Šamajová O. Vadovič P. Pechan T. Košútová P. Ovečka M. (2014). Proteomic and biochemical analyses show functional network of proteins involved in antioxidant defense of *Arabidopsis anp2anp3* double mutant. *J. Prot. Res.* 13 5347–5361. 10.1021/pr500588c 25325904 PMC4423761

[B69] Takahashi M. Asada K. (1988). Superoxide production in aprotic interior of chloroplast thylakoids. *Arch. Biochem. Biophys.* 267 714–722. 10.1016/0003-9861(88)90080-x 2850770

[B70] Toruño T. Y. Shen M. Coaker G. Mackey D. (2019). Regulated disorder: posttranslational modifications control the RIN4 plant immune signaling hub. *Mol. Plant Microbe Interact.* 32 56–64. 10.1094/MPMI-07-18-0212-FI 30418084 PMC6501815

[B71] Tsang C. K. Liu Y. Thomas J. Zhang Y. Zheng X. F. S. (2014). Superoxide dismutase 1 acts as a nuclear transcription factor to regulate oxidative stress resistance. *Nat. Commun.* 5:3446. 10.1038/ncomms4446 24647101 PMC4678626

[B72] Valasatava Y. Rosato A. Banci L. Andreini C. (2016). MetalPredator: a web server to predict iron–sulfur cluster binding proteomes. *Bioinformatics* 32 2850–2852. 10.1093/bioinformatics/btw238 27273670

[B73] Van Breusegem F. Slooten L. Stassart J. M. Moens T. Botterman J. Van Montagu M. (1999). Overproduction of *Arabidopsis* thaliana FeSOD confers oxidative stress tolerance to transgenic maize. *Plant Cell Physiol.* 40 515–523. 10.1093/oxfordjournals.pcp.a029572 10427774

[B74] Van Camp W. Capiau K. Van Montagu M. Inzé D. Slooten L. (1996). Enhancement of oxidative stress tolerance in transgenic tobacco plants overproducing Fe-superoxide dismutase in chloroplasts. *Plant Physiol.* 112 1703–1714. 10.1104/pp.112.4.1703 8972606 PMC158104

[B75] Waszczak C. Carmody M. Kangasjärvi J. (2018). Reactive oxygen species in plant signaling. *Annu. Rev. Plant Biol.* 69 209–236. 10.1146/annurev-arplant-042817-040322 29489394

[B76] Waters B. M. McInturf S. A. Stein R. J. (2012). Rosette iron deficiency transcript and microRNA profiling reveals links between copper and iron homeostasis in *Arabidopsis thaliana*. *J. Exp. Bot.* 63 5903–5918. 10.1093/jxb/ers239 22962679 PMC3467300

[B77] Wong P. K. (2000). Effects of 2,4-D, glyphosate and paraquat on growth, photosynthesis and chlorophyll-a synthesis of *Scenedesmus quadricauda* Berb 614. *Chemosphere* 41 177–182. 10.1016/s0045-6535(99)00408-7 10819198

[B78] Xiong Y. Contento A. L. Nguyen P. Q. Bassham D. C. (2007). Degradation of oxidized proteins by autophagy during oxidative stress in *Arabidopsis*. *Plant Physiol.* 143 291–299. 10.1104/pp.106.092106 17098847 PMC1761971

[B79] Yamasaki H. Hayashi M. Fukazawa M. Kobayashi Y. Shikanai T. (2009). SQUAMOSA promoter binding protein-like7 is a central regulator for copper homeostasis in *Arabidopsis*. *Plant Cell* 21 347–361. 10.1105/tpc.108.060137 19122104 PMC2648088

[B80] Zhang J. Vancea A. I. Shahul Hameed U. F. Arold S. T. (2021). Versatile control of the CDC48 segregase by the plant UBX-containing (PUX) proteins. *Comput. Struct. Biotechnol. J.* 19 3125–3132. 10.1016/j.csbj.2021.05.025 34141135 PMC8181520

