# Supplementary material for: Arabidopsis Iron Superoxide Dismutase FSD1 Protects Against Methyl Viologen-Induced Oxidative Stress in a Copper-Dependent Manner
Source: Front Plant Sci. 2022 Mar 11;13:823561. doi: 10.3389/fpls.2022.823561 (PMC8963501; doi:10.3389/fpls.2022.823561)
Supplement: Supplementary file 8 [file Image_1.pdf]

Supplementary Material

Arabidopsis iron superoxide dismutase FSD1 protects against methyl viologen-induced oxidative stress in a copper-dependent manner

Pavol Melicher, Petr Dvořák, Yuliya Krasylenko, Alexey Shapiguzov, Jaakko Kangasjärvi, Jozef Šamaj, Tomáš Takáč

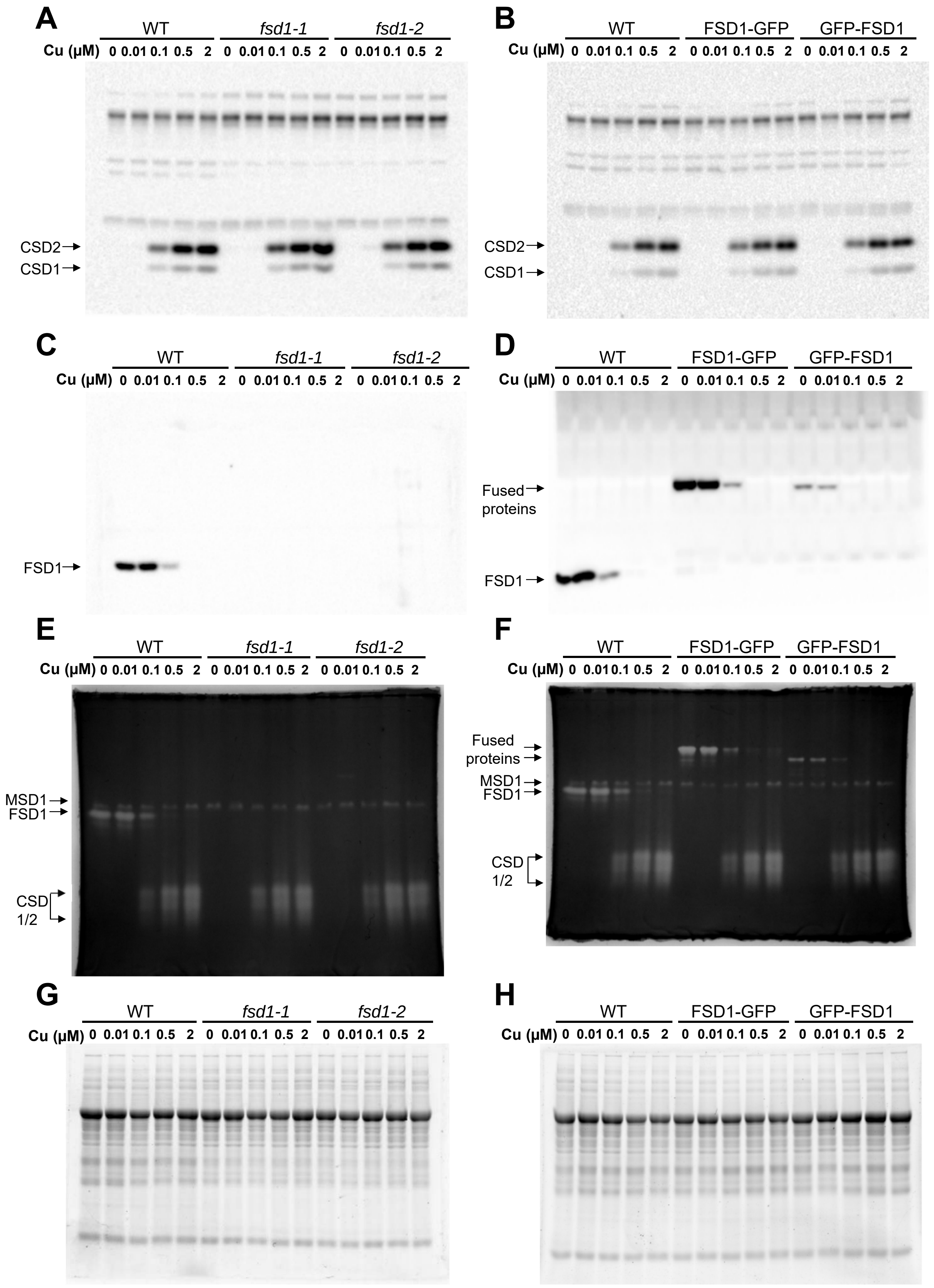

Supplementary Figure 1 Superoxide dismutases (SODs) abundances and activities in wild type (WT), *fsd1-1, fsd1-2* mutants and transgenic plants growing on different concentrations of copper (0 μM - 2 μM) in growing media. **(A-D)** Immunoblots of CSD1 and CSD2 (A, B) and FSD1 (C, D) in WT, *fsd1-1*, *fsd1-2* mutants (A, C) and in FSD1-GFP and GFP-FSD1 lines (B, D). **(E, F)** SOD activity staining in WT, *fsd1-1*, *fsd1-2* mutants (E) and in FSD1-GFP and GFP-FSD1 lines (F). **(G, H)** Respective controls of protein loading as visualized on Stain-free gels.

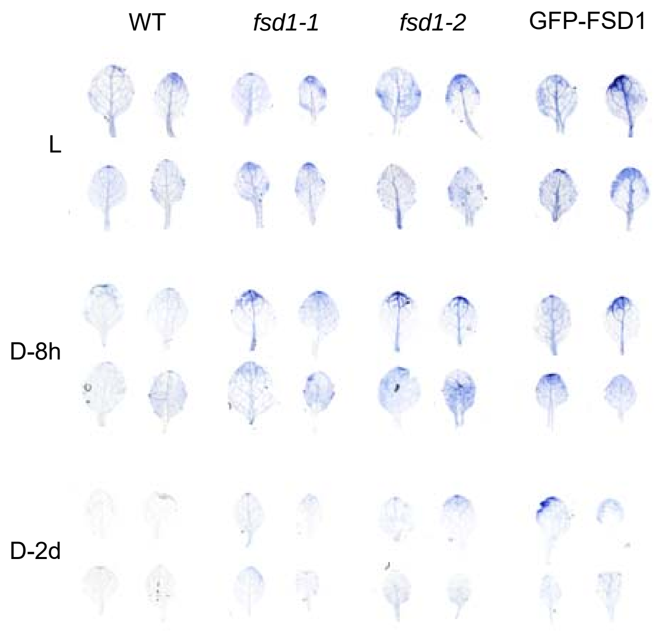

Supplementary Figure 2 Histochemical evaluation of superoxide ($O_2^{\bullet-}$) in leaves of 10-day-old *Arabidopsis* seedlings of wild type (WT), *fsd1-1*, *fsd1-2* mutants and GFP-FSD1 line. $O_2^{\bullet-}$ production was visualized as dark blue coloration by nitroblue tetrazolium staining in seedlings incubated under normal light conditions (L) or in dark for 2 days (D-2d) or 8 h (D-8h).

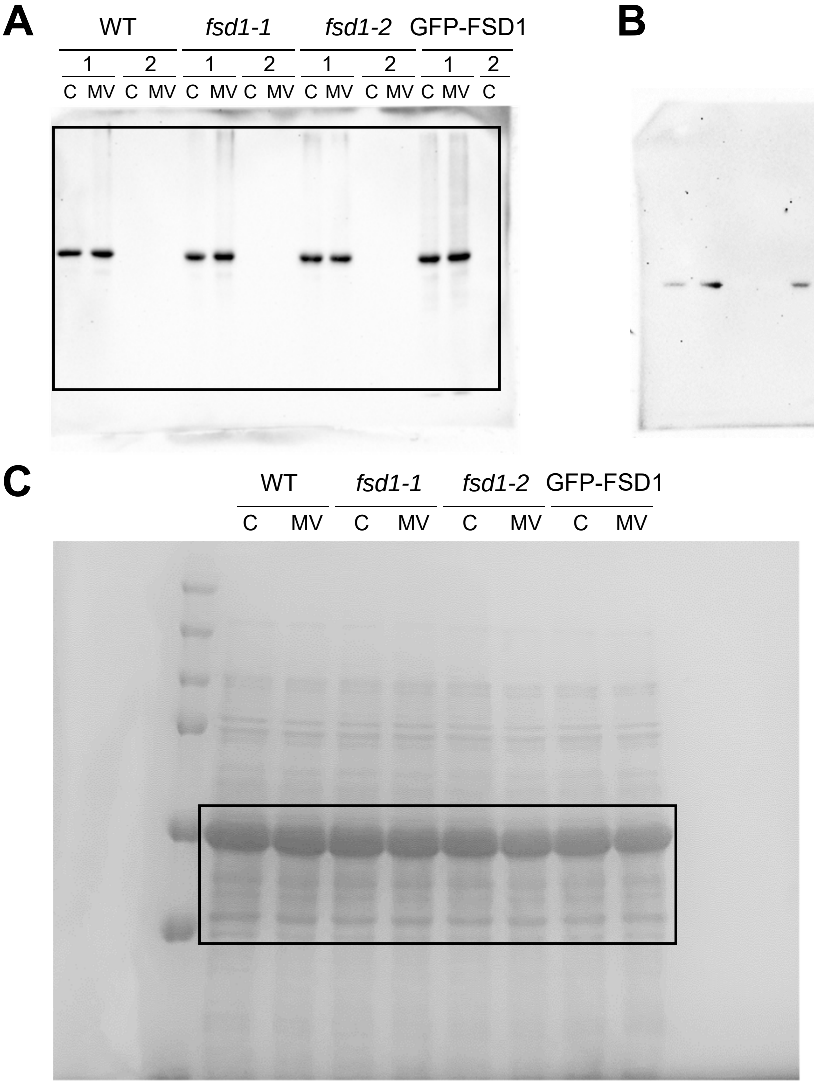

Supplementary Figure 3 Full scan of the entire original blots and Comassie Briliant Blue G-250-stained gel presented in Figure 4B,D. **(A, B)** Entire membranes with chemiluminiscent signal observed after probing with anti-DNP antibody included in OxyBlot™ kit. Each blot contains protein extracts from mock (lane C) and 1 μM MV treated (lane MV) plants. Each sample was treated either with DNPH oxidation reagent (1) or a control reagent (2) included in the OxyBlot kit. **(B)** The MV-treated sample of GFP-FSD1 derivatized with control reagent was run on a separate gel B followed by the same procedure applied for A. Samples loaded on lanes which are not annotated are not relevant to this study. The highlighted regions show the presented sections in Figure 4B. **(C)** Entire Comassie Briliant Blue G-250-stained gel. The highlighted region shows the presented section in Figure 4D.

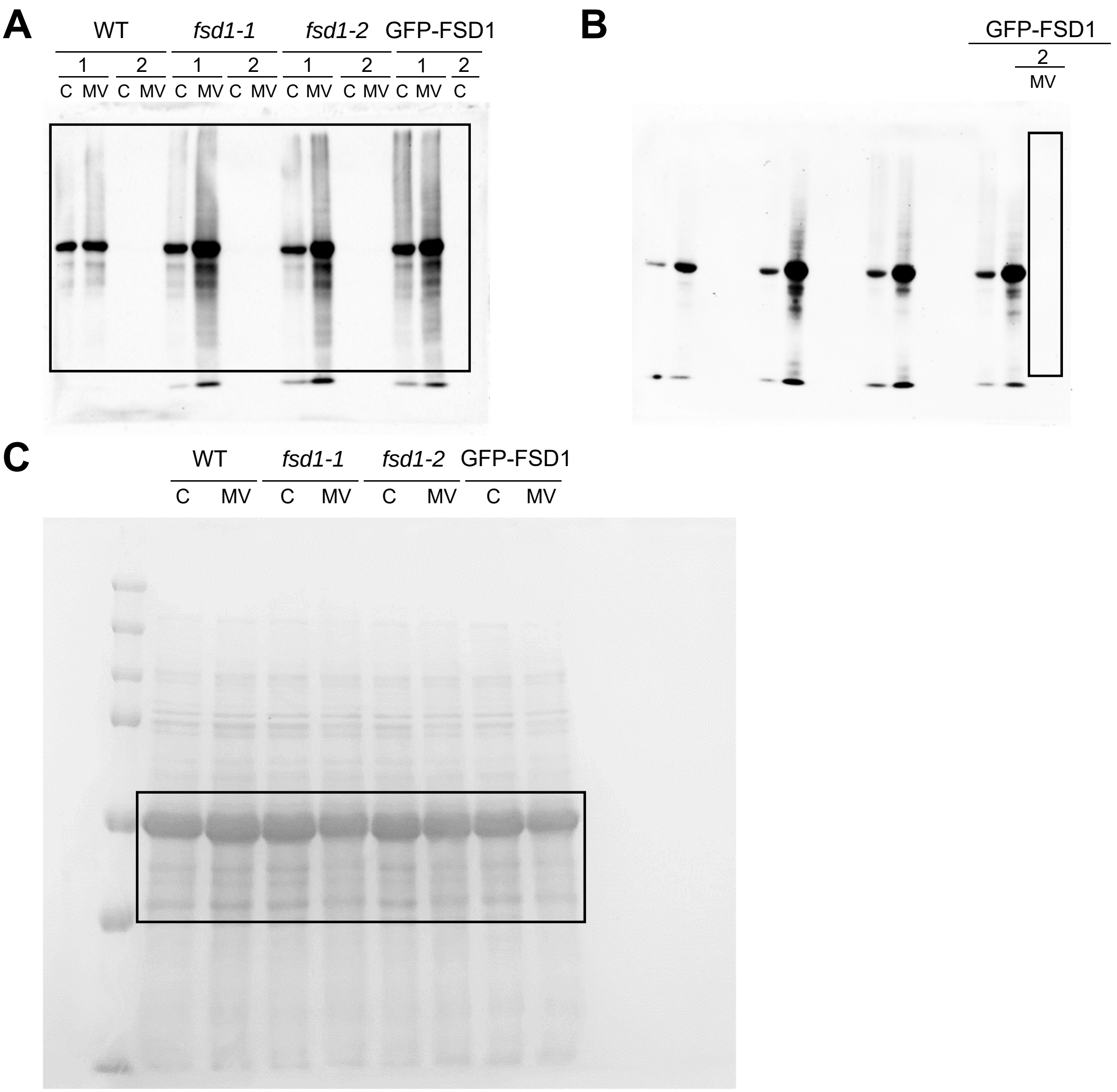

Supplementary Figure 4 Full scan of the entire original oxyblots and Comassie Briliant Blue G-250-stained gel presented in Figure 4C,E. **(A, B)** Entire membranes with chemiluminiscent signal observed after probing with anti-DNP antibody included in OxyBlot™ kit. Each blot contains protein extracts from mock (lane C) and 1 µM MV treated (lane MV) plants. Each sample was treated either with DNPH oxidation reagent (1) or a control reagent (2) included in the OxyBlot kit. **(B)** The MV-treated sample of GFP-FSD1 derivatized with control reagent was run on a separate gel B followed by the same procedure applied for membrane A. Samples loaded on lanes which are not annotated are not relevant to this study. The highlighted regions show the presented sections in Figure 4C. **(C)** Entire Comassie Briliant Blue G-250-stained gel. The highlighted region shows the presented section in Figure 4E.

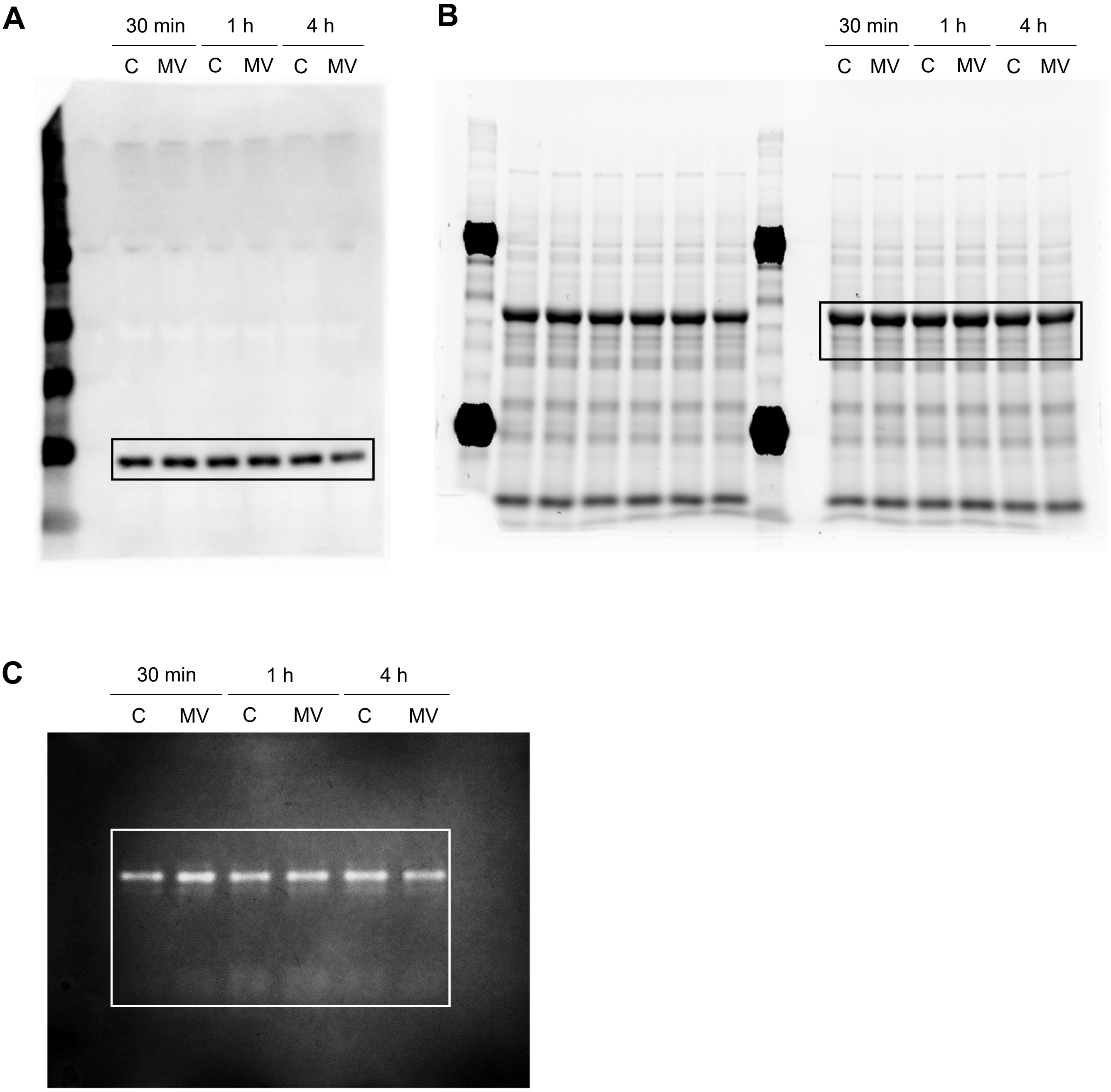

Supplementary Figure 5 Full scan of the entire original immunoblot and gel stained for specific activity of superoxide dismutases (SODs) presented in Figure 5A and 5C. Results related to study of wild type (WT) in response to 1 µM methyl viologen (MV) are shown. **(A)** Entire membrane with chemiluminiscent signal observed after probing with anti-FSD antibody. **(B)** Full image of the Stain-free gel with separated proteins representing equal amount of proteins loaded on gel prior to transfer to polyvinylidene difluoride membrane. The highlighted regions show the sections presented in Figure 5A. Samples loaded on lanes which are not annotated are not relevant to this study. **(C)** Full scan of the entire original gel stained for specific activity of SODs. The highlighted region shows the presented section in Figure 5C. The membrane (A) and gels (B, C) contain protein extracts from mock control conditions (lane C) and 1 µM MV treatment (lane MV) treated for 30 min, 1 h and 4 h.

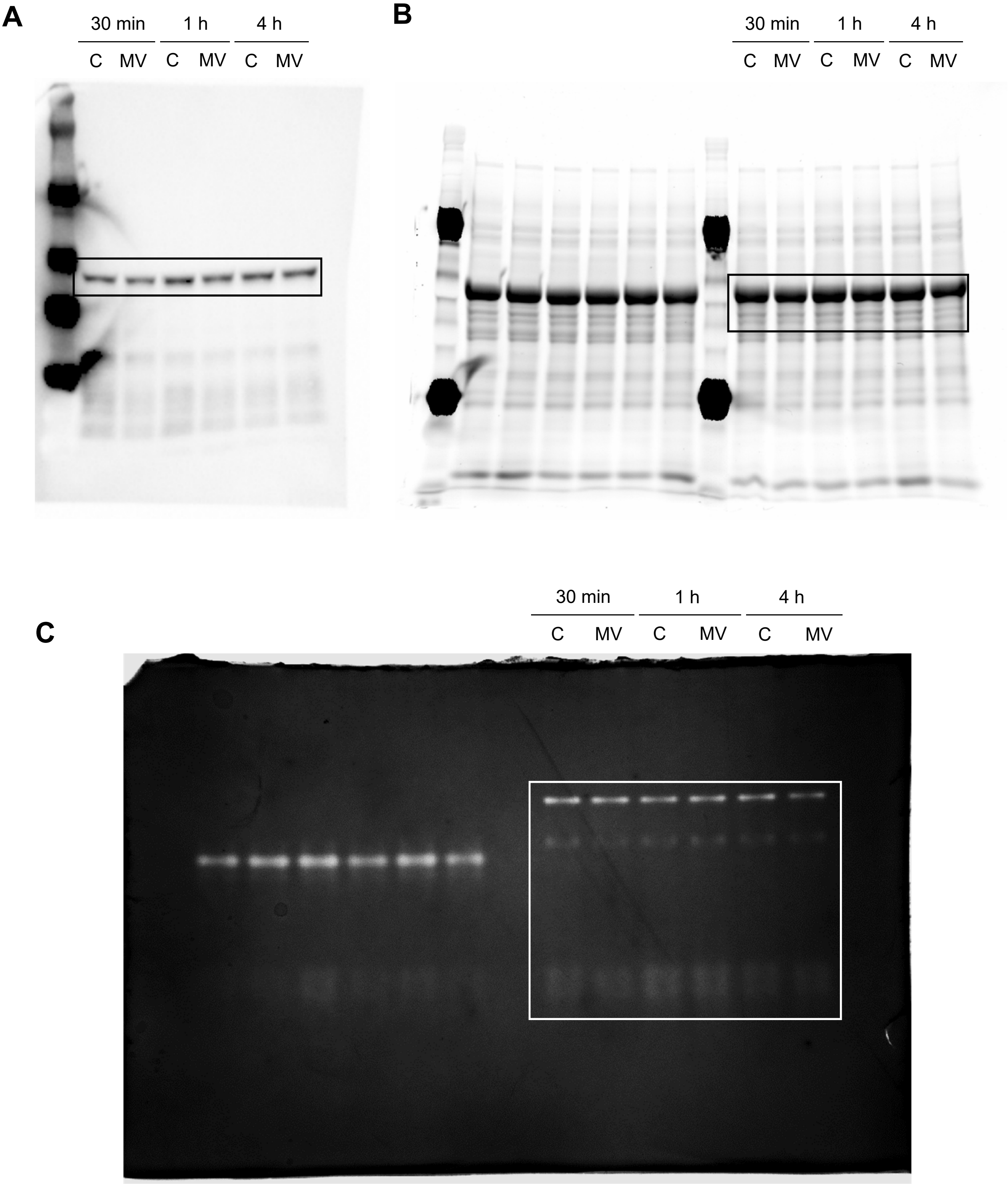

Supplementary Figure 6 Full scan of the entire original immunoblot and gel stained for specific activity of superoxide dismutases (SODs) presented in Figure 5E and 5G. Results related to study of GFP-FSD1 line in response to 1 µM methyl viologen (MV) are shown. **(A)** Entire membrane with chemiluminiscent signal observed after probing with anti-FSD antibody. **(B)** Full image of the Stain-free gel with separated proteins representing equal amount of proteins loaded on gel prior to transfer to polyvinylidene difluoride membrane. The highlighted regions show the sections presented in Figure 5E. Samples loaded on lanes which are not annotated are not relevant to this study. **(C)** Full scan of the entire original gel stained for specific activity of SODs. The highlighted region shows the presented section in Figure 5G. The membrane (A) and gels (B, C) contain protein extracts from mock control conditions (lane C) and 1 µM MV treatment (lane MV) treated for 30 min, 1 h and 4 h.

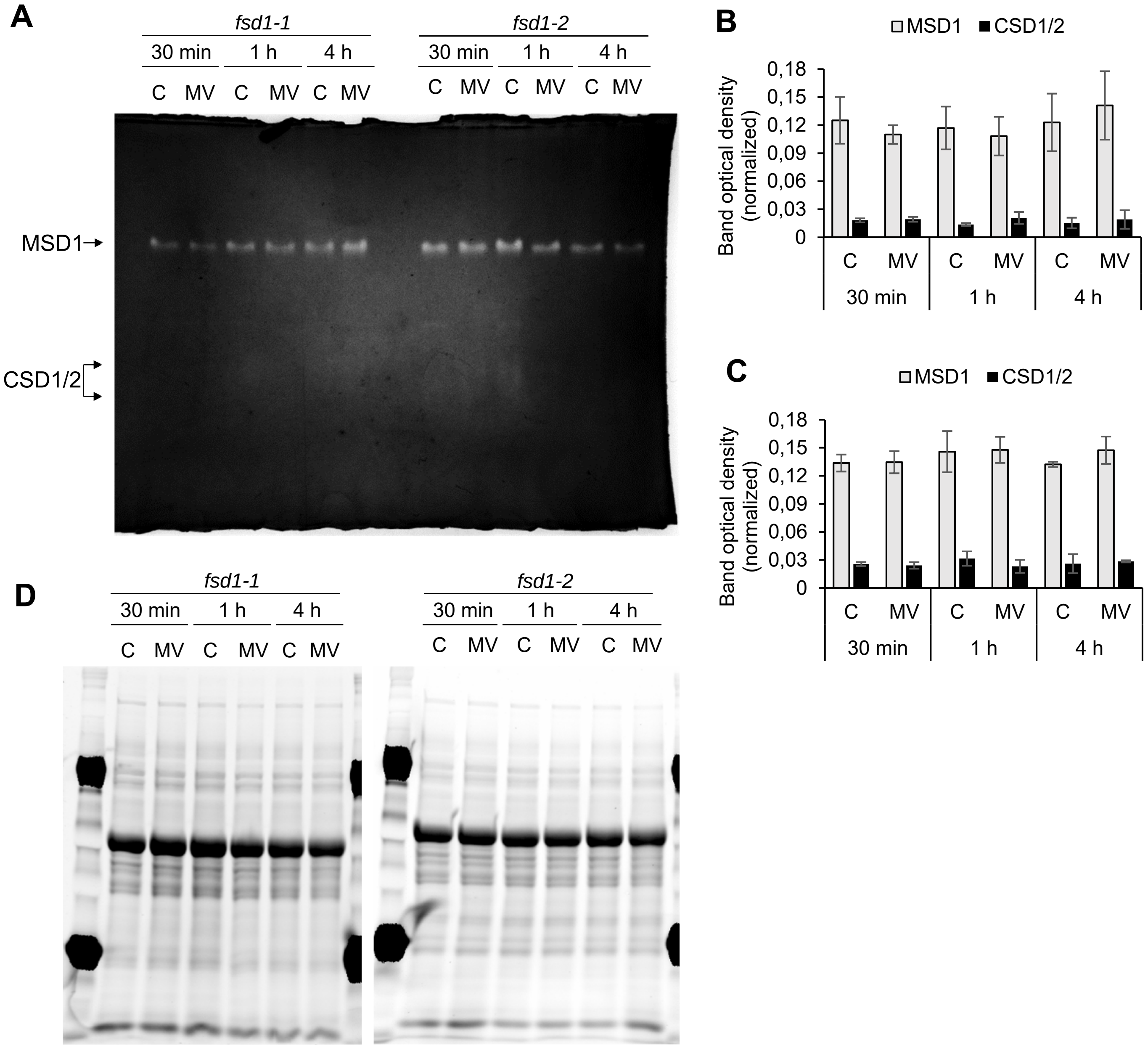

Supplementary Figure 7 Superoxide dismutase (SOD) activity in *fsd1-1* and *fsd1-2* mutants in response to methyl viologen (MV). **(A)** SOD activity staining on native PAGE gels in *fsd1-1* and *fsd1-2* mutants supplemented with respective controls of protein loading **(D)** as visualized on Stain-free gels. Each gel contains protein extracts from mock (lane C) and 1 µM MV-treated (lane MV) plants. **(B, C)** Quantification of band optical density in A for *fsd1-1* (B) and *fsd1-2* (C) mutants. Data was normalized according to the total density of the specific bands on the membrane (mean ± SD, N = 3).

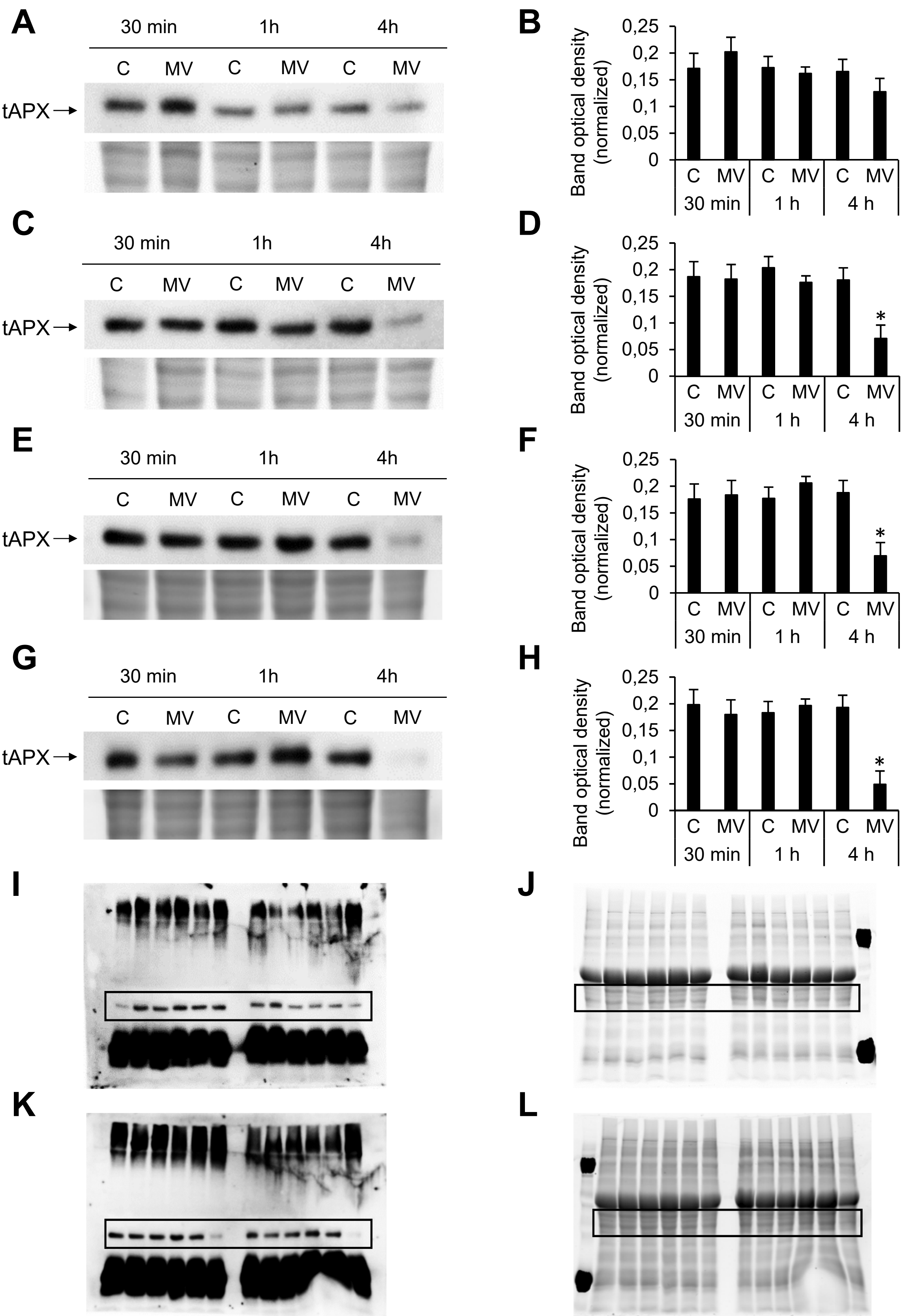

Supplementary Figure 8 Analysis of thylakoid ascorbate peroxidase (tAPX) abundance in wild type (WT), *fsd1-1*, *fsd1-2* mutants and GFP-FSD1 lines after methyl viologen (MV) treatment. **(A, C, E, G)** Immunoblots of tAPX in WT (A), *fsd1-1* (C), *fsd1-2* (E) mutants and GFP-FSD1 line (G), supplemented with respective controls of protein loading as visualized on Stain-free gels. Each immunoblot contains protein extracts from mock (lane C) and 1 µM MV treated (lane MV) plants. **(B, D, F, H)** Quantification of band optical density in A, C, E, G. Data was normalized according to the total density of the specific bands on the membrane (mean ± SD, N = 3). Asterisks indicate a statistically significant difference between mock and 1 µM MV treated plants in designated time points as calculated by one-way ANOVA with post-hoc Tukey HSD test ($p < 0.05$). **(I, J, K, L)** Uncropped, full original images of the blots and gels of both WT, *fsd1-1* (I, J) and *fsd1-2*, GFP-FSD1 (K, L). The highlighted regions show the presented sections.

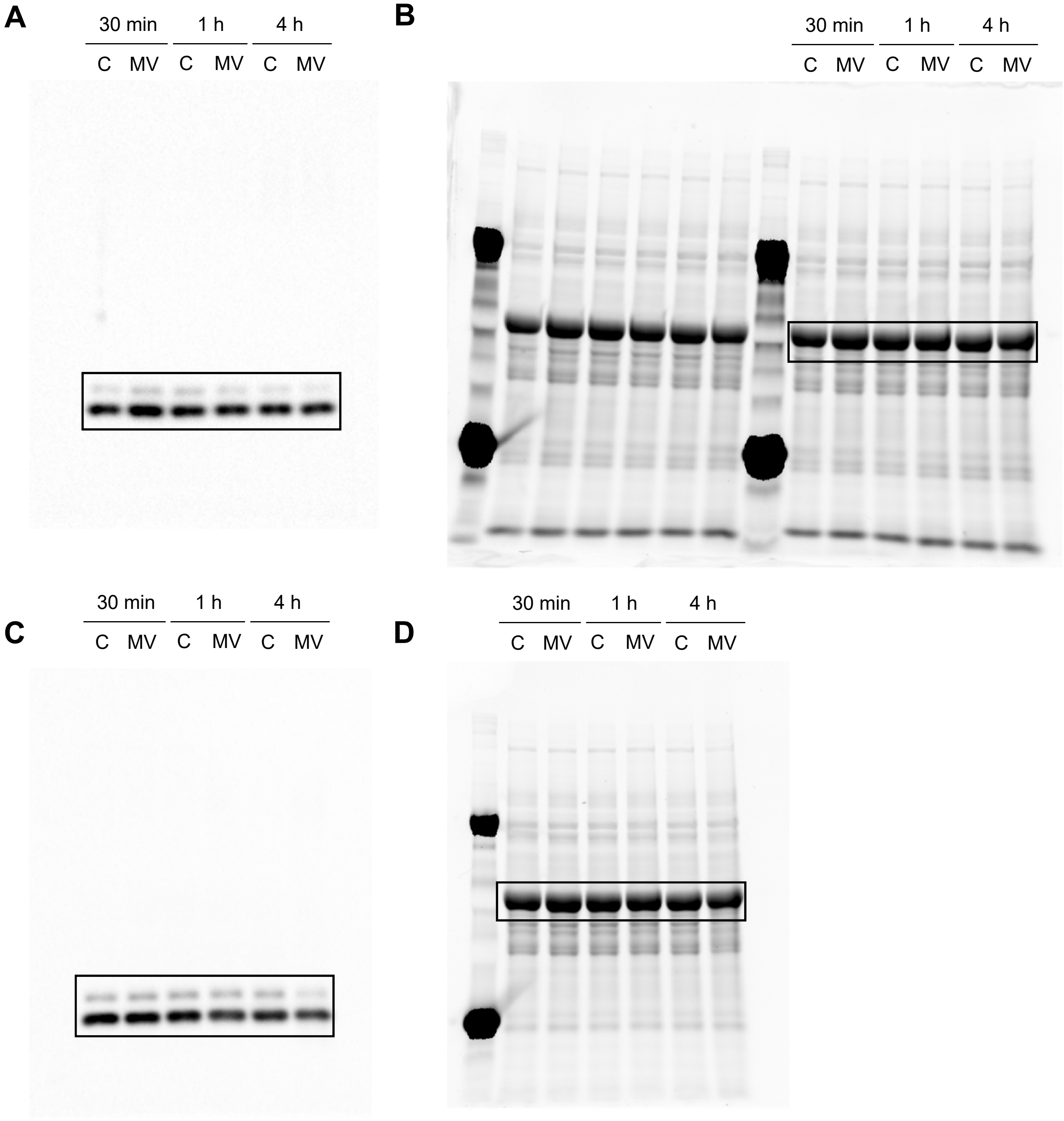

Supplementary Figure 9 Full scan of the entire original immunoblots presented in Figures 6B and 6D. Results related to wild type (WT; A, B) and *fsd1-1* mutant (C, D) in response to 1 µM methyl viologen (MV) are shown. **(A, C)** Entire membrane with chemiluminiscent signal observed after probing with anti-APX antibody. **(B, D)** Full image of the Stain-free gel with separated proteins representing equal amount of proteins loaded on gel prior to transfer to polyvinylidene difluoride membrane. The highlighted regions show the sections presented in Figures 6B (A, B) and 6D (C, D). Samples loaded on lanes which are not annotated are not relevant to this study. Membranes (A, C) and gels (B, D) contain protein extracts from mock control conditions (lane C) and 1 µM MV treatment (lane MV) treated for 30 min, 1 h and 4 h.

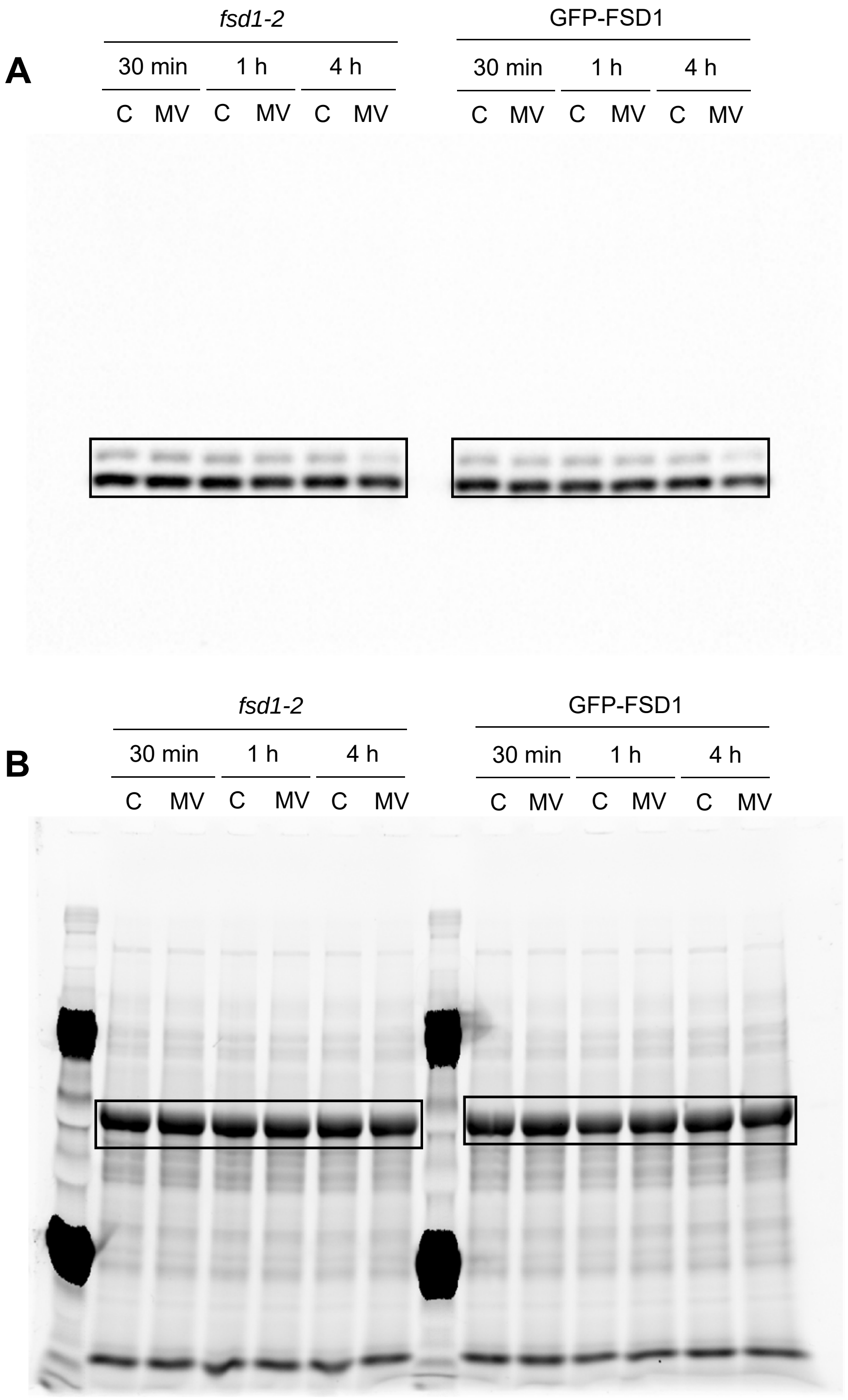

Supplementary Figure 10 Full scan of the entire original immunoblot presented in Figures 6F and 6H. Results related to *fsd1-2* mutant and GFP-FSD1 line in response to 1 µM methyl viologen (MV) are shown. **(A)** Entire membrane with chemiluminiscent signal observed after probing with anti-APX antibody. **(B)** Full image of the Stain-free gel with separated proteins representing equal amount of proteins loaded on gel prior to transfer to polyvinylidene difluoride membrane. The highlighted regions show the sections presented in Figures 6F (A) and 6H (B). Samples loaded on lanes which are not annotated are not relevant to this study. Membrane (A) and gel (B) contain protein extracts from mock control conditions (lane C) and 1 µM MV treatment (lane MV) treated for 30 min, 1 h and 4 h.

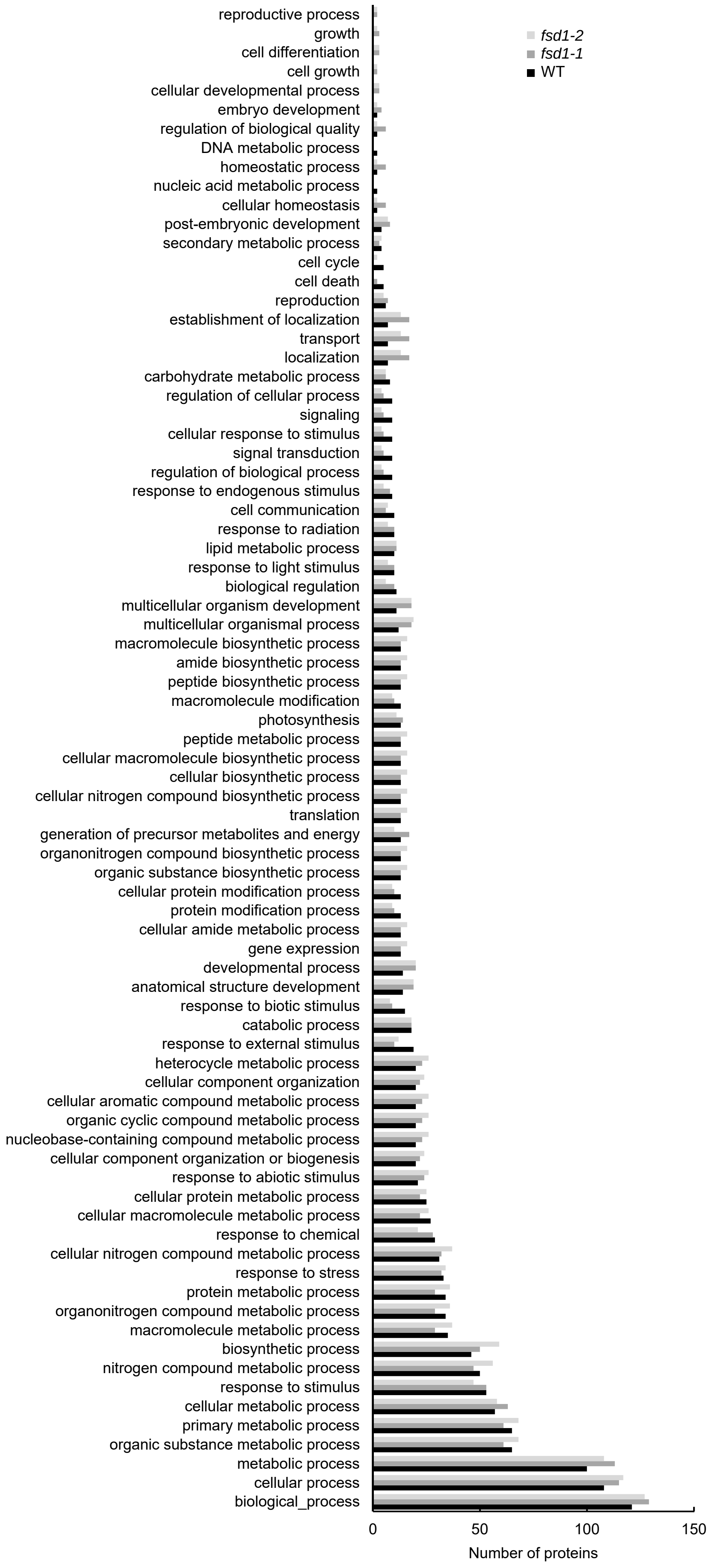

Supplementary Figure 11 Gene ontology annotation of differential proteomes which were retrieved by comparison of methyl viologen-treated plants (wild type (WT), *fsd1-1* and *fsd1-2* mutants) with mock-treated plants, according to biological process.

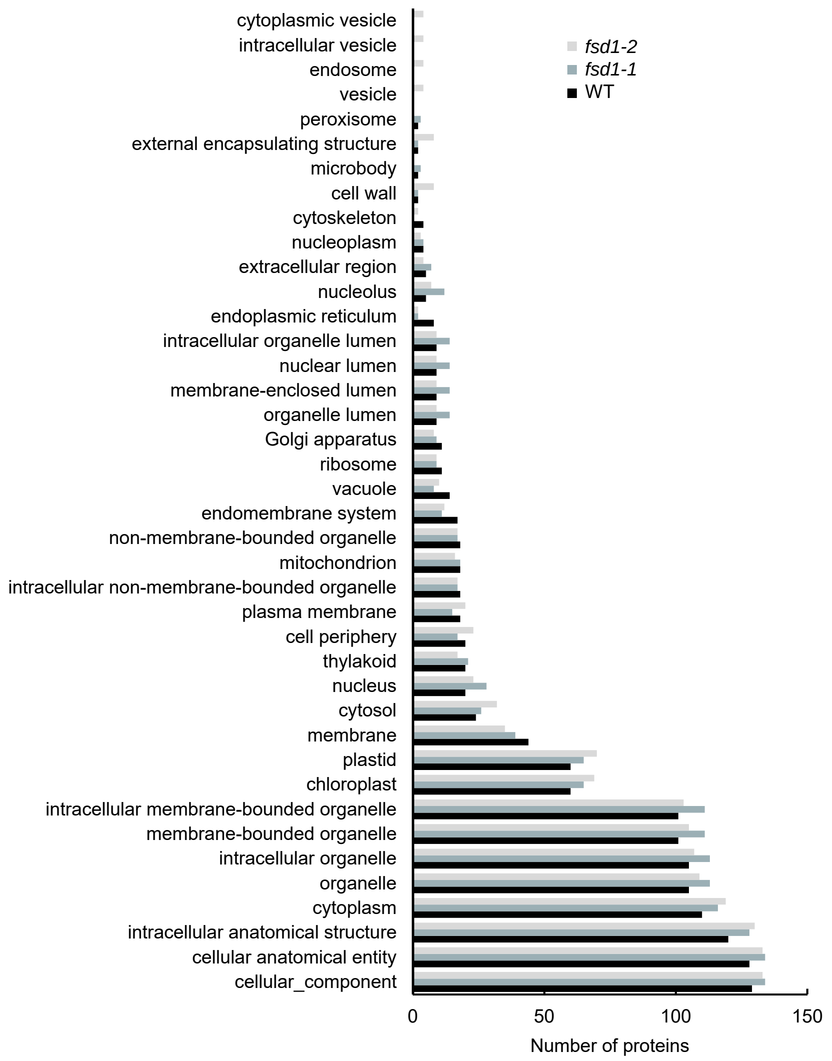

Supplementary Figure 12 Gene ontology annotation of differential proteomes which were retrieved by comparison of methyl viologen-treated plants (wild type (WT), *fsd1-1* and *fsd1-2* mutants) with mock-treated plants, according to cell compartment.

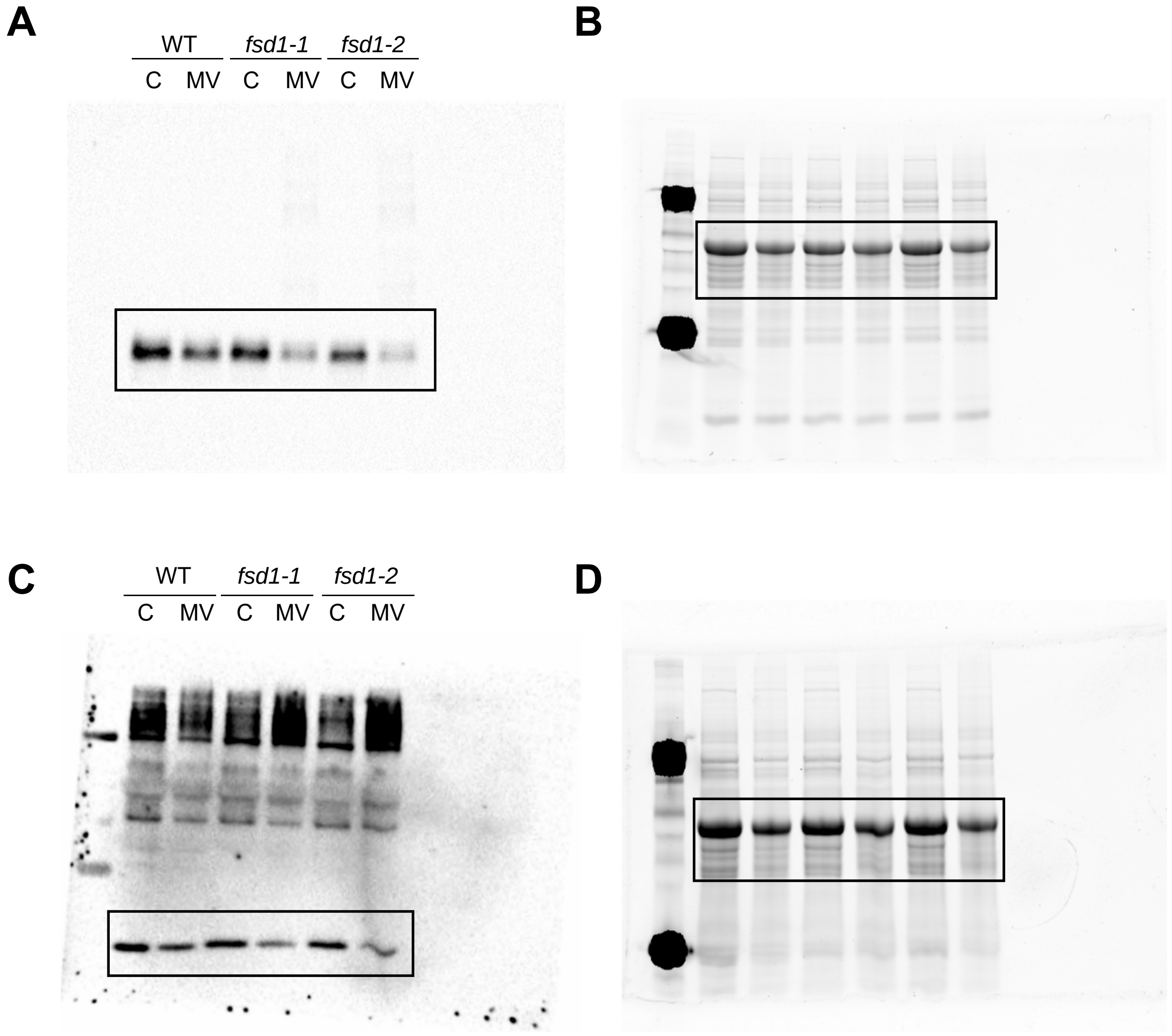

Supplementary Figure 13 Full scan of the entire original immunoblots presented in Figure 8. **(A)** Entire membrane with chemiluminiscent signal observed after probing with anti-ferredoxin 2 antibody. **(B)** Respective controls of protein loading as visualized on Stain-free gel. The highlighted regions show the sections presented in Figure 8A. **(C)** Entire membrane with chemiluminiscent signal observed after probing with anti-ferritis antibody. **(D)** Respective controls of protein loading as visualized on Stain-free gel. The highlighted regions show the sections presented in Figure 8C.
